# Chronic sleep restriction in the rotenone Parkinson’s disease model in rats reveals peripheral early-phase biomarkers

**DOI:** 10.1038/s41598-018-37657-6

**Published:** 2019-02-13

**Authors:** Juliane Fagotti, Adriano D. S. Targa, Lais S. Rodrigues, Ana Carolina D. Noseda, Flávia W. C. Dorieux, Franciele F. Scarante, Jessica L. Ilkiw, Fernando M. Louzada, Namrata R. Chowdhury, Daan R. van der Veen, Benita Middleton, Jeroen L. A. Pennings, Jonathan R. Swann, Debra J. Skene, Marcelo M. S. Lima

**Affiliations:** 1 Department of Physiology, Federal University of Paraná, Curitiba, PR Brazil; 2 Chronobiology, Faculty of Health and Medical Sciences, University of Surrey, Guildford, UK; 3 RIVM - National Institute for Public Health and the Environment, Bilthoven, The Netherlands; 4 Division of Computational and Systems Medicine, Department of Surgery and Cancer, Faculty of Medicine, Imperial College London, South Kensington, London, UK

## Abstract

Parkinson’s disease (PD) is a chronic disorder that presents a range of premotor signs, such as sleep disturbances and cognitive decline, which are key non-motor features of the disease. Increasing evidence of a possible association between sleep disruption and the neurodegenerative process suggests that sleep impairment could produce a detectable metabolic signature on the disease. In order to integrate neurocognitive and metabolic parameters, we performed untargeted and targeted metabolic profiling of the rotenone PD model in a chronic sleep restriction (SR) (6 h/day for 21 days) condition. We found that SR combined with PD altered several behavioural (reversal of locomotor activity impairment; cognitive impairment; delay of rest-activity rhythm) and metabolic parameters (branched-chain amino acids, tryptophan pathway, phenylalanine, and lipoproteins, pointing to mitochondrial impairment). If combined, our results bring a plethora of parameters that represents reliable early-phase PD biomarkers which can easily be measured and could be translated to human studies.

## Introduction

Parkinson’s disease (PD) is a chronic neurodegenerative disease that typically affects dopaminergic neurons in the substantia nigra pars compacta (SNpc). However, other regions such as brainstem nuclei, cortical areas, spinal cord, preganglionic sympathetic/parasympathetic neurons, as well as portions of the peripheral and enteric nervous systems are involved in the pathophysiology^1–4^. Before occurrence of the prominent motor signs, PD presents a range of non-motor symptoms (NMS) that precede the clinical motor phase by many years. Some are well-known, such as olfactory and gastrointestinal dysfunction, sleep disorders, circadian changes and cognitive impairment^3,5–7^. Moreover, neuropathological studies support the association of these early-phase disturbances based on the identification of Lewy bodies in non-dopaminergic nuclei in early Braak stages, prior to significant SNpc degeneration and motor signs^2^. Recent epidemiological studies propose that NMS can appear up to 25 years before the onset of clinical PD^6^, and it is well-established that patients report sleep disruption at least a decade before the first motor symptoms^8^. In animal models, the SNpc was shown to regulate sleep patterns^9^ and recently it was found that sleep-wake disturbance can predispose the brain to PD neuropathology^10^. Undoubtedly, sleep disorders represent an essential part of PD progression, once brain structures affected in the first stages of the disease^11^ and correspondent neurotransmitter systems are involved in sleep regulation^12^, but they are poorly investigated in the diagnosis.

Reduced total sleep time, sleep efficiency and sleep fragmentation, all leading to sleep loss, consistently emerge as sleep issues in PD^13,14^, but it is unclear if sleep loss constitutes a risk-factor for PD due to the lack of more specific prospective studies^15^. In general, these sleep alterations are one of the premotor features that most affect the patients’ quality of life, and could contribute to worsening cognitive abilities, such as memory impairment^16,17^, apart from having a direct association with the motor impairment^18^. In this context, PD-related sleep disturbances^5^ and society-imposed sleep restrictions^19^ may contribute to cognitive decline, and even emerge as an early biomarker of abnormal aging^20^, perhaps producing detectable changes in peripheral tissues in addition to behavioural parameters, like memory deficits and circadian shifts. Despite much effort, there is as yet no reliable way to identify those individuals that will develop PD. Failure to establish the pathological process is the main obstacle to find a cure or treatment that alters the course of the disease, but our inability to diagnose it early enough hinders a better approach or improvement of the existing treatments. Therefore, the identification of risk factors and detection of early symptoms are a priority, since no approach to date has identified specific or sensitive signs that have a practical application in diagnosis^21,22^. Metabolic phenotyping (metabonomics/metabolomics) using high resolution analytical chemistry platforms coupled with multivariate statistics provides great potential for identifying reliable biomarkers of PD. The elucidation of such biochemical signatures could represent a major step towards early diagnosis, disease progression, and effective treatments^23,24^.

Here we investigated, in the rotenone (ROT) animal model of PD, chronic sleep restriction (SR) as a possible triggering factor for peripheral metabolic changes, cognitive impairment and circadian alterations. Rotenone, a mitochondrial complex I activity inhibitor pesticide, mimics the hallmark traits of early-phase PD (equivalent to Braak stages 2–3) extending to NMS such as excessive daytime sleepiness, REM sleep behaviour disorder, insomnia and deterioration of spontaneous sleep, dopamine-dependent behavioural deficits, hyposmia and gastrointestinal problems, as well as canonical pathological alterations, such as time-dependent reduction of dopaminergic nigrostriatal neurons, α-synuclein (PARK1) aggregation, Lewy-like body formation, oxidative stress and ultrastructural impairments in the SNpc mitochondria^5,25–35^. To identify the biochemical perturbations associated with SR in this model, two metabolic profiling platforms were applied to characterize the plasma metabolic signatures: global ^1^H nuclear magnetic resonance (NMR) spectroscopy and targeted liquid chromatography/mass spectrometry (LC/MS).

## Methods

### Ethics Approval

All animal procedures were approved by the Ethics Committee of the Federal University of Paraná (approval ID #858) and conducted according to the guidelines of ethics and experimental care and use of laboratory animals (SBCAL). The research protocols were reviewed by The University of Surrey Animal Welfare Ethical Review Body (AWERB) and by the Named Veterinary Surgeon (NVS) prior to any work, in accordance with the principles of UK legislation (Animals [Scientific Procedures] Act 1986).

### Experimental design

The experiments were performed on healthy male Wistar rats, 90 days old, weighing 280–330 g, with normal immune status. Experimental animals were not involved in any previous test or drug treatment. The animals were group housed (4 per cage) in polypropylene cages with disposable bedding on a standard light-dark (L/D) cycle (12 h:12 h cycle, lights on at 07.00 h = ZT0) in a temperature controlled room (22 ± 2 °C). Food and water were provided *ad libitum* throughout the experiment.

**Figure 1:**
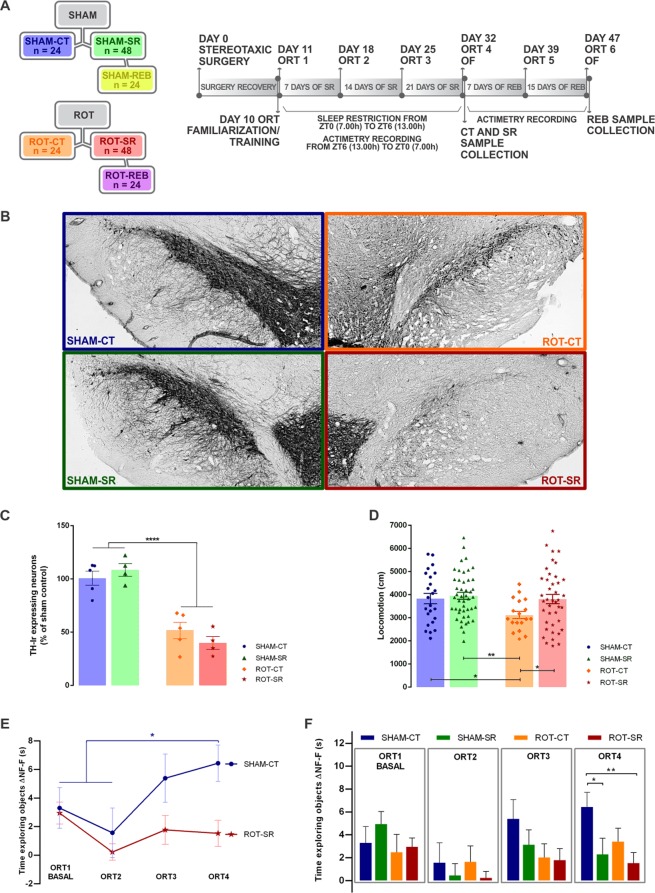
Sleep restriction in the rotenone model of PD. (**A**) Group distribution and timeline diagram. Colours are representative of groups throughout the results. SR = Sleep Restriction; REB = Rebound sleep; SHAM = vehicle; ROT = rotenone; SHAM-CT = sham control for SR (blue); ROT-CT = rotenone control for SR (orange); SHAM-SR = vehicle SR (green); ROT-SR = rotenone SR (red); SHAM-REB = SHAM-SR after 15 days of REB (yellow); ROT-REB = ROT-SR after 15 days of REB (purple); ORT = Object Recognition Task; OF = Open Field. (**B**) Representative immunohistochemistry labelling of TH-ir neurons at the end of SR for SHAM-CT, ROT-CT, SHAM-SR and ROT-SR. (**C**) Percentage of TH-ir expressing neurons in the SNpc in relation to the SHAM-CT group. Individual data are shown as a scatter plot with bar of mean ± standard error of the mean (SEM), n = 4 per group for CT (SHAM-CT and ROT-CT) and n = 5 per group for SR (SHAM-SR and ROT-SR). ****P ≤ 0.0001 sham compared to rotenone treated (two-way ANOVA followed by Fisher’s LSD test). (**D**) Locomotion parameter obtained from the open field test. Individual data (cm) are shown as a scatter plot with bar of mean ± SEM. *P < 0.05; **P ≤ 0.01 (two-way ANOVA followed by Fisher’s LSD test). n = 24 SHAM-CT; n = 46 SHAM-SR; n = 20 ROT-CT; n = 41 ROT-SR. (**E**) Exploration index (ΔNF–F). Bars represent the mean (±SEM) time exploring objects (s). *P < 0.05 (two-way ANOVA followed by Fisher’s LSD test). ORT 1 = First test, before SR; ORT 2 = second test (SR = 7 days); ORT 3 = third test (SR = 14 days); ORT 4 = fourth test (SR = 21 days). n = 24 SHAM-CT; n = 24 SHAM-SR; n = 20 ROT-CT; n = 20 ROT-SR. NF = non-familiar object; F = familiar object. (**F**) Learning curve comparing SHAM-CT and ROT-SR during the SR protocol. Time exploring objects (ΔNF–F) values (s) are expressed as mean ± SEM. *P < 0.05; **P ≤ 0.01 (one-way repeated-measures ANOVA followed by Fisher’s LSD test). n = 24 SHAM-CT; n = 20 ROT-SR.

Figure 1A shows the group distribution flowchart and a timeline diagram of the study design. The animals were randomly distributed in one of the groups: SHAM-CT (vehicle infusion and control for sleep restriction)/SHAM-SR (vehicle infusion and sleep restriction condition)/SHAM-REB (vehicle infusion and sleep restriction condition followed by sleep recovery)/ROT-CT (rotenone infusion and control for sleep restriction)/ROT-SR (rotenone infusion and sleep restriction condition)/ROT-REB (rotenone infusion and sleep restriction condition followed by sleep recovery). Total of 6 groups, n = 24 animals per group, total number of 144 animals. The experimental design was performed in 6 cohorts (6 groups with 4 animals each, total number of 24 animals per cohort).

One week before the experiments began, the animals were maintained in the room described above for habituation. On day 0, the animals underwent stereotaxic surgery. After a 10 day interval for recovery, the animals were trained for the Object Recognition Task (ORT) and the following day the basal test (ORT 1) was performed prior to 21 days of sleep restriction (commencing on day 11). Every week, ORT was repeated on the same weekday (ORT 2 = 7 days of SR; ORT 3 = 14 days of SR) until the end of sleep restriction (day 32 = 21 days of SR). On day 32, another ORT (ORT 4) was carried out, followed by an Open Field Test and then the control (CT) groups (SHAM-CT; ROT-CT) and half of the animals from the SR groups (SHAM-SR, n = 24, and ROT-SR, n = 24) were sacrificed. The other half of the SHAM-SR (n = 24) and ROT-SR (n = 24) groups were allowed sleep recovery for 15 days (Rebound group (REB) – SHAM-REB and ROT-REB) at the end of the sleep restriction period. Throughout the experiment, animals’ activity was recorded daily for 21 days by infrared motion sensors after the 6 h of sleep restriction and throughout the rebound period.

### Stereotaxic surgery

The animals were initially sedated with intraperitoneal xylazine (10 mg/kg; Syntec do Brasil Ltda, Brazil) and anaesthetized with ketamine (90 mg/kg, i.p.; Syntec do Brasil Ltda, Brazil). For ROT (3 groups, 24 animals per group, total number of 72 animals) or vehicle (dimethylsulfoxide - SHAM) (3 groups, 24 animals per group, total number of 72 animals) infusion within the SNpc, the following coordinates was used, bregma as a reference: (AP) −5.0 mm, (ML) ± 2.1 mm and (DV) −8.0 mm^36^. Rotenone (12 µg/µl; Sigma-Aldrich®, St. Louis, MO, United States) or DMSO (Sigma-Aldrich®, St. Louis, MO, United States) infusions of 1 µL into each hemisphere were made at a rate of 0.33 µL/min for 3 min^32,33,37^. These infusions were made using an electronic infusion pump (Insight Instruments, Ribeirão Preto, SP, Brazil).

### Sleep restriction procedure

SR (4 groups, 24 animals per group, total number of 96 animals) was performed using a gentle handling/stimulation protocol, which consisted of soft tapping on the cage, gently shaking the cage or, when this was not sufficient to keep animals awake, gently disturbing the sleeping nest^38^. CT animals (2 groups, 24 animals per group, total number of 48 animals) were left undisturbed.

### Open Field (OF) Test

The OF test apparatus consisted of a circular arena (1 m diameter) limited by a 50 cm high wall. The animals were placed in the centre of the arena and allowed to freely explore the area for 5 min. The animals’ distance travelled was recorded by a digital camera coupled to the Smart Junior system (Panlab, Harvard Apparatus Spain, Barcelona, Spain)^39^.

### Object Recognition Task

The ORT was performed in an open box (80 cm (W) × 80 cm (L) × 50 cm (H)) made of wood and covered with a black, opaque plastic film in dim light conditions. The objects to be discriminated were made of different materials, all of them previously covered with non-toxic varnish, so that the animals could not move them around in the arena. The objects are not known to have any ethological significance for the rats and had never been associated with reinforcement^40^. The first day of the experiment consisted of 4 familiarization/training trials (3 min each, 15–20 min apart), in which two identical objects (familiar objects) were placed in the back corners of the open box, 10 cm away from the sidewall. The rat was placed in the open box facing away from the objects. Twenty-four hours after training, the first test of choice was performed (ORT 1), where one of the familiar objects was replaced by a new object (non-familiar for the animal). The test was repeated every seven days, when the non-familiar object was always replaced and the familiar one remained the same, totalling four tests for the animals that experienced daily sleep restriction only or control (ORT 2: 7 days after ORT 1; ORT 3: 14 days after ORT 1; ORT 4: 21 days after ORT 1) and six ORT tests for the animals that remained in rebound after the end of the sleep restriction period (ORT 5: 28 days after ORT 1; ORT 6: 36 days after ORT 1). The time spent exploring each object was analysed by the same experimenter, blind to animal condition, to avoid operator bias. Exploration was defined as only occurring when the rat touched the object with its nose or the rat’s nose was directed towards an object at a distance ≤2 cm. The familiar/non-familiar object locations (left or right side of the arena) were counterbalanced within each experiment, as well as within-subject for subsequent experiments. The arena and objects were cleaned between each trial with 70% ethanol^41^. The results were calculated using the time spent by rats exploring the familiar (a′) and the non-familiar object (b) (Δ = b-a′)^40^.

### Diurnal activity measurement

Overall activity of all animals in a cage was measured continuously after the sleep restriction and throughout the rebound period. Home cage activity was continuously recorded by passive infrared detectors connected to a computer that registered the motion (in-house apparatus). The device recorded the amount of activity in seconds in 5-minutes bins. Raw data were primarily analysed with the in-house software CTools 8.0.

### TH-immunohistochemistry

For TH-immunoreactive (TH-ir) neurons quantification, the animals were anaesthetized with ketamine. Then, each animal was transcardially perfused with a saline solution, followed by a fixative solution of formaldehyde 4% in 0.1 M phosphate buffer (pH 7.4). After that, brains were removed from the skulls and were immersed in the fixative solution at 4 °C. Forty-eight hours later, the material was immersed in a 30% sucrose solution for three days and finally stored in a −80 °C freezer before sectioning. Nine 40 µm sections corresponding to SNpc (−4.92 mm and −5.28 mm/coordinates obtained from Paxinos and Watson^36^) were collected from 5 animals of each group. Three slices were randomly chosen from each animal and incubated with primary mouse anti-TH antibody (1:500; Chemicon, Rolling Meadows, IL, USA). Biotin-conjugated secondary antibody (1:200 anti-mouse; Vector Laboratories, Burlingame, CA, USA), was localized using the ABC system (Vectastain ABC Elite kit, Vector Laboratories, Burlingame, CA, USA), followed by 3,30-diaminobenzidine reaction with nickel enhancement. Neuronal density determination was conducted using the software Image J (National Institutes of Health, Rockville, MD, USA). For each group, a mean value was calculated and converted to a percentage relative to the sham control group and compared with the other groups (data not shown for REB groups, which had no statistical differences to the correspondent SR group). The images were obtained using a motorized Axio Imager Z2 microscope (Carl Zeiss, Jena, Germany), equipped with an automated scanning VSlide (Metasystems, Altlussheim, Germany).

### Sample collection for metabolomics

For metabolomics analyses, plasma samples were collected after decapitation. At this stage, brains were also removed from the skulls and structures of interest (SNpc, striatum and hippocampus) were dissected for later analysis. Following this, 10 mL of blood was placed into lithium heparin tubes, which were gently inverted 5–8 times. The samples were centrifuged immediately at 3200 rpm (1620 g) for 10 minutes at 4 °C and split into aliquots (1 mL and 25 µl). The plasma aliquots were placed immediately into a −80 °C freezer. The samples were stored at −80 °C prior to being shipped on dry ice to the UK (University of Surrey/Imperial College) for LC/MS or NMR metabolomics analysis.

### Targeted metabolomics analysis (LC/MS)

Plasma samples were measured using the AbsoluteIDQ® p180 targeted metabolomics kit (Biocrates Life Sciences AG, Innsbruck, Austria), and a Waters Xevo TQ-S mass spectrometer coupled to an Acquity UPLC system (Waters Corporation, Milford, MA, USA) similar to our previous studies^42–44^. Plasma samples (10 μl) were prepared according to the manufacturer’s instructions adding several stable isotope–labelled standards to the samples prior to the derivatization and extraction steps. Using either LC/MS or flow injection analysis/MS up to 183 metabolites from 5 different compound classes (namely acylcarnitines, amino acids, biogenic amines, glycerophospholipids and sphingolipids) can be quantified. All the plasma samples and 3 levels of quality control (QC) were processed on a single 96-well plate, sample order being randomised. The levels of metabolites present in each QC were compared to the expected values and the percent coefficient of variation (CV%) calculated. Metabolites where >25% concentrations were below the limit of detection (<LOD) or below lower limit of quantification (≪LLOQ) or above limit of quantification (>LOQ) or blank out of range, or the QC2 coefficient of variance was >30%, were excluded (n = 50)^43,44^. The remaining 133 quantified metabolites comprised 10 acylcarnitines, 21 amino acids, 12 biogenic amines, 76 glycerophospholipids and 14 sphingolipids.

### ¹H NMR spectroscopy

Plasma samples were prepared as described by Beckonert, *et al*.^45^. ^1^H NMR spectroscopy was performed at 310 K on a Bruker 600 MHz spectrometer (Bruker Biospin, Karlsruhe, Germany). For each sample, water-suppressed Carr-Purcell-Meiboom-Gill spin-echo spectra were recorded. In this experiment, eight dummy transients were followed by 64 transients and collected in 64 K data points. NMR spectra were manually corrected for phase and baseline distortions and referenced to the anomeric proton of β-glucose at δ 5.223.

### Quantification and Statistical Analysis

Animals were randomly assigned to experimental groups, with no specific randomization strategy. The criteria for calculating the sample size was established from the formula that establishes the sample size for a finite population: *n* = *[N*. *σ²*. *(Z*_*α/2*_*)²]/[(N-1)*.*E² + σ²*. *(Z*_*α/2*_*)²]*, where n = number of individuals; Zα/2 = critical value that corresponds to the desired confidence interval; E = the maximum error of the estimate; N = population size; σ = population standard deviation. Thus, the following equation was used for this study: *N* = *10*. *52*. *(1*.*96) 2*/*(10-1) 0*.*052* + *52*. *(1*.*96) 2* = *9*.*99 ≅* 10 (sample number per group/parameter). Consequently, it was stipulated that each group should comprise a sample number of 10 rats for each analysis.

For behavioural data, statistical analysis was performed using GraphPad Prism Version 6.0 (GraphPad Software, La Jolla, CA, USA) and Statistica Version 12 (StatSoft Inc, Tulsa, OK, USA). Values were presented as mean ± SEM. Homogeneity of variance was assessed by the Bartlett test and normal distribution of the data was assessed by the Kolmogorov-Smirnov test. Statistical significance for behavioural results was set at P < 0.05. Data inclusion was determined by The ROUT method of identifying outliers (Q = 10%) for the Open Field Test and by the Box Plot Diagram to identify outliers for the Object Recognition Task, where an outlier was that with a value more than 1.5 times the interquartile range above the third quartile or below the first quartile (animals with 3 or more values excluded from the ORT tests, outlier or data acquisition issues, had all values excluded).

Statistical analysis of TH-immunohistochemistry, Open Field Test, Object Recognition Test and Rest-activity pattern was performed using two-way ANOVA followed by Fisher’s LSD test, using treatment (SHAM/ROT) and procedure (CT/SR/REB) as independent variables. For the learning curve of Object Recognition Test, a one-way repeated-measures ANOVA followed by Fisher’s LSD test was performed using the Δ (Δ = b-a′) of each group.

LC/MS metabolomics analysis: multivariate analysis was performed by principal component analysis (PCA) and orthogonalized partial least squares discriminant analysis (OPLS-DA), using SIMCA-P v13.0 software (Umetrics, Umeå, Sweden) and default software settings. Whereas PCA looks at overall variation (unsupervised), OPLS-DA distinguishes between class-predictive (discriminating) variation (supervised) and non-predictive (orthogonal) variation. The use of these methods allows insight into the extent and underlying patterns of class-predictive variation within the data set as a whole. Differences in individual metabolite levels were analysed in R version 3.1.2 (R Foundation for Statistical Computing, Vienna, Austria) using the linear models and ANOVA methods in the stats package. Linear models were fitted to the treatment (SHAM/ROT) and procedure (CT/SR/REB). Significant differences for all parameters and their interaction were determined using multivariate ANOVA. P-values were corrected for multiple comparisons according to the Benjamini-Hochberg False Discovery Rate (FDR). Metabolites were considered as significant at an FDR cutoff < 0.05. For testing statistical significance, missing values (n = 15) were not taken into account. For data visualisation, we used a heat map combined with hierarchical clustering (Euclidean distance and Ward linkage). Prediction models based on combinations of metabolites were made by logistic modelling in R statistical software. First, metabolites were selected that met the following criteria: a significant (FDR < 0.05) difference between the control and group of interest in the ANOVA model; a (non-cross-validated) Area Under the Curve (AUC) that was significantly higher than for randomly permutated values (P < 0.05, determined by 10,000 permutations); and a difference in plasma concentration of more than 5% between groups. For the markers that met these criteria, logistic models were made using log-transformed values. The predictive performance of the models was tested by determining the AUC using leave-one-out cross-validation, in which a model was trained using data of all-but-one and tested on the remaining rat. Using this approach, models were developed by stepwise testing the result of adding another metabolite to the current model and selecting the metabolite with the highest gain in AUC to be included in the model for the next step. This was repeated until no further improvement in AUC could be achieved. For the models obtained, predictions were shown by Receiver Operating Characteristic (ROC) visualisation. Finally, for determining sensitivity and specificity of the SR models, samples with a predicted probability of more than 50% were considered predicted as SR, whereas samples with a predicted probability of less than 50% were considered predicted as not-SR.

NMR spectroscopy: Spectra were digitized using an in-house MATLAB (version R2009b, The Mathworks, Inc.; Natick, MA, USA) script. The spectral region containing the water resonance was excised from the spectra. PCA was then performed with Pareto scaling in Matlab^46^. Overview PCA analysis identified 4 outliers in the data set (1 due to high plasma lactate; 3 had high plasma glucose). These samples were removed from subsequent analyses, including OPLS-DA analysis performed using in-house scripts in Matlab.

## Results

### Rotenone lesion combined with Sleep Restriction altered locomotor activity, lead to cognitive impairment and delay of rest-activity rhythm

Characterization of dopaminergic neuronal loss by TH immunohistochemistry in the SNpc (representative photomicrographs Fig. 1B) showed that Rotenone (ROT) (12 µg/µl) infusion (1 µl at 0.33 µl/min) induced a decrease of TH-ir neurons when compared to the sham groups (SHAM-CT vs. ROT-CT = 49% reduction; SHAM-CT vs. ROT-SR = 61% reduction; SHAM-SR vs. ROT-CT = 57% reduction; SHAM-SR vs. ROT-SR = 69% reduction), [F(1,14) = 74.58; P < 0.0001] (Fig. 1C). SR did not have a significant effect overall. The percentage reduction of TH-ir neurons was maintained after 15 days of sleep recovery (REB) (Fig. S1A).

In order to evaluate the integrity of the locomotor activity we analysed the animals’ performance on Open Field Test. As can be seen in Fig. 1D, the ROT-CT group, on day 32, exhibited a decrease in the distance travelled in the OF compared with all other groups on the same day, with an effect both of the lesion [F (1,125) = 4.415; P < 0.05] and of the sleep restriction [F(1,125) = 3.964; P < 0.05]. After REB, there was no statistical difference between the groups (Fig. S1B).

We used the Object Recognition Test – a measure where the time spent on a new object presented to the animal permits to evaluate recognition memory – to estimate the cognition status. The sham CT group exhibited a significant learning curve as shown by the memory improvement over subsequent tasks [F (83.544) = 3.129; P < 0.05], with a marked difference between ORT1/2 and ORT4 (P < 0.05) (Fig. 1E). In each test, a two-way ANOVA followed by Fisher’s LSD test between groups was performed revealing that in the last test (ORT4) SR impaired both SHAM (P < 0.05) and ROT (P < 0.01) groups compared to SHAM-CT (Fig. 1F), which was the only group that explored the non-familiar object significantly more after ORT2 (Fig. S2). Moreover, in the first week of sleep rebound, significant memory impairment in the ROT-REB group compared to SHAM-REB (P < 0.05) was observed. However, after 15 days of sleep rebound, this difference between the groups disappeared (Fig. S1C).

**Figure 2:**
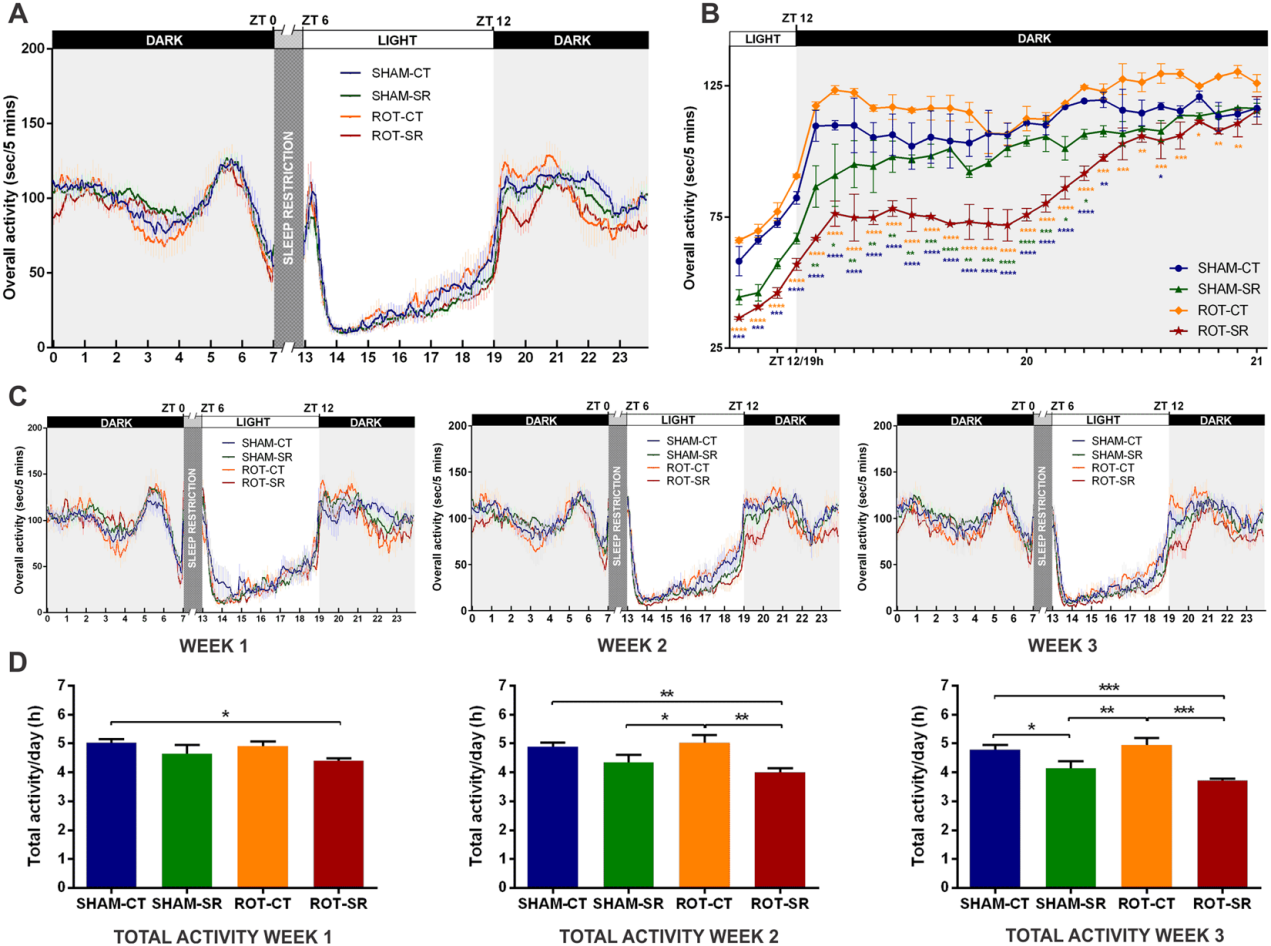
Rest-activity parameters. (**A**) Daily activity pattern of each group (seconds/5 minutes) during the sleep restriction (SR) period. Each line represents the mean activity (±SEM) across the 21 days of SR. ZT0 = lights on and the beginning of gentle handling until ZT6 when recording started. n = 6 per group for CT (SHAM-CT and ROT-CT) and n = 12 per group for SR (SHAM-SR and ROT-SR). (**B**) Activity profile 15 minutes before and 2 hours after the onset of darkness (ZT12) during weeks 2 and 3 after the start of SR. Each line represents the mean activity (seconds/5 minutes) of the 14 days (±SEM). Ticks on x axis represent 5-minutes blocks. *P < 0.05; **P ≤ 0.01; ***P ≤ 0.001; ****P ≤ 0.0001 (two-way ANOVA followed by Fisher’s LSD test). Orange (*) = ROT-CT compared to ROT-SR; Blue (*) = ROT-SR compared to SHAM-SR; Green (*) = ROT-SR compared to SHAM-SR. n = 6 per group for CT (SHAM-CT and ROT-CT) and n = 12 per group for SR (SHAM-SR and ROT-SR). (**C**) Daily activity pattern each week of the 3 weeks of SR. Each line represents the mean activity (seconds/5 minutes) of 7 days ± SEM. ZT0 - lights on and the beginning of gentle handling until ZT6 when recording started. n = 6 per group for CT (SHAM-CT and ROT-CT) and n = 12 per group for SR (SHAM-SR and ROT-SR). (**D**) Total activity/day (h) in each of the 3 weeks of SR. The bars represent the mean ± SEM. *P < 0.05; **P ≤ 0.01; ***P ≤ 0.001 (two-way ANOVA followed by Fisher’s LSD test). n = 24 SHAM-CT; n = 24 SHAM-SR; n = 20 ROT-CT; n = 20 ROT-SR.

The assessment of the activity pattern to observe possible rest-activity alterations was performed using an infrared beam-break system. The two-way ANOVA analysis revealed that there was a statistical difference in the diurnal activity profile among groups [F (3,6912) = 38.56; P < 0.0001] at several time-points [F (215,6912) = 87.72; P < 0.0001] over the 21 days of SR (Fig. 2A). There was a consistent diminished activity level in the ROT-SR group starting at ZT12 (lights-off; 19:00 h clock time) that remained lower for at least 1 hour after the onset of darkness. This difference was not seen in the first week but emerged and became statistically significant in weeks 2 and 3 (Fig. 2C). We investigated the first 2 hours of the dark phase in the course of weeks 2 and 3 in more detail, and this analysis showed that the ROT-SR group exhibited significantly reduced activity [F (3,100) = 220.4; P < 0.0001] during the 75 minutes after lights-off, suggesting reduced behavioural arousal (Fig. 2B). Total activity also showed a progressive decline each week in the SR groups: in week 1 [F (1,83) = 4.753; P < 0.05] only ROT-SR showed a significant difference from SHAM-CT (P < 0.05); in the second week both SR groups showed reduced activity [F (1,83) = 12.94; P < 0.001] that was even more evident in week 3 [F (1,83) = 19.46; P < 0.0001] (Fig. 2D). On the first day after SR ended, both SHAM and ROT groups exhibited a disturbed rest-activity rhythm throughout the day, which recovered over time (Fig. S3).

### Plasma metabolic profiling

**Figure 3:**
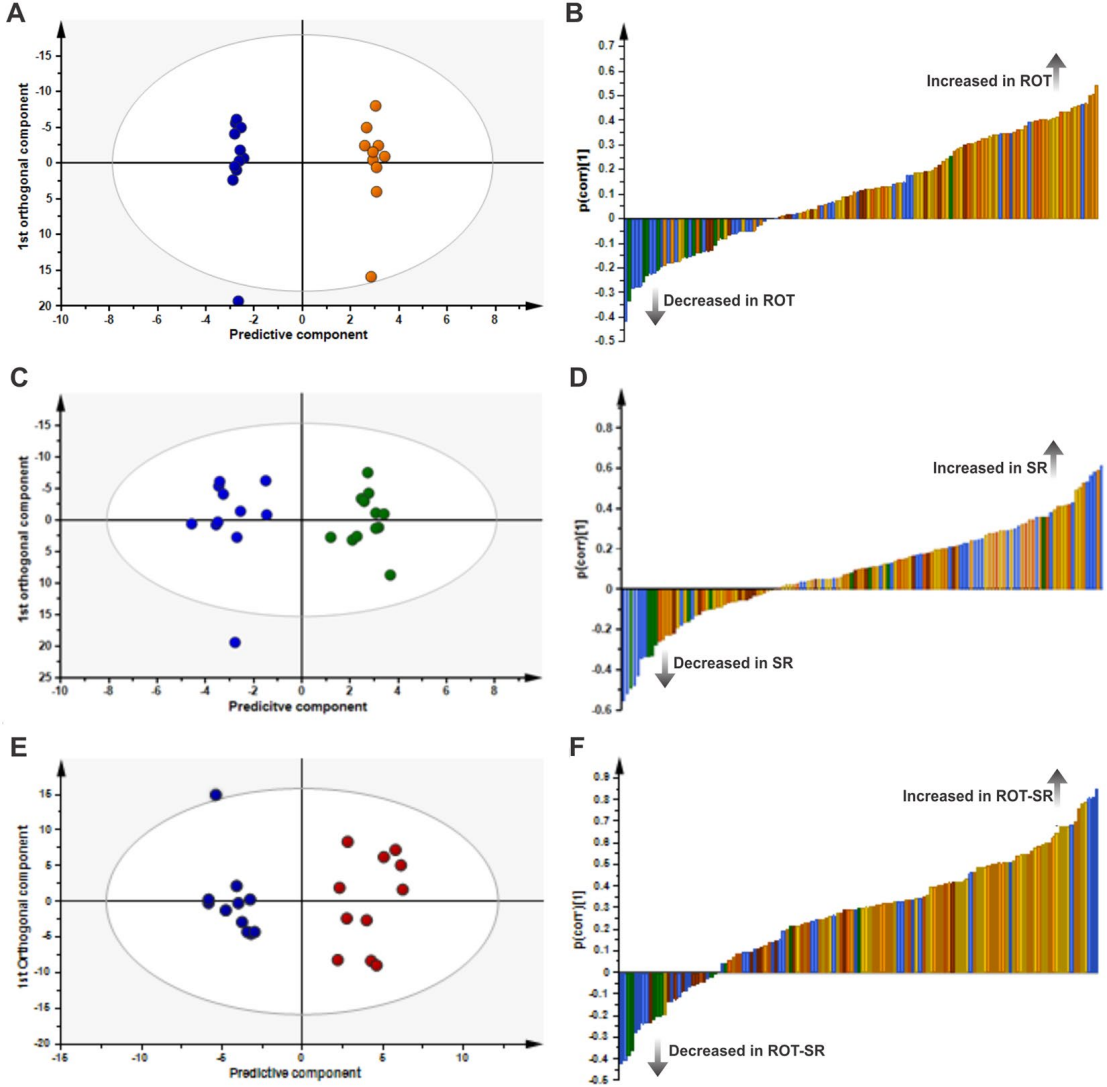
OPLS-DA plots in LC/MS targeted metabolomics data. (**A**) OPLS-DA scores plot showing separation by phenotype between the sham control group (SHAM-CT, dark blue, n = 11) and the rotenone control (ROT-CT, orange, n = 10); R^2^X 0.636; R^2^Y 0.996; Q²Y 0.312; P < 0.05. (**B**) OPLS-DA loading plot of SHAM-CT vs ROT-CT. Negative p(corr) values represent decreased and positive p(corr) values represent increased metabolite concentrations in the rotenone group (ROT-CT) compared to sham group (SHAM-CT). The metabolite bars are colour coded according to metabolite class as follows: amino acids and biogenic amines (blue); acylcarnitines (green); lysophosphatidylcholine acyl (lyso PC a) (dark orange); phosphatidylcholine diacyl (PC aa) (yellow); phosphatidylcholine acyl-alkyl (PC ae) (light orange); sphingolipids (SM) (brown). (**C**) OPLS-DA scores plot showing separation by sleep restriction alone (blue, SHAM-CT, n = 11; green, SHAM-SR, n = 12); R^2^X 0.421; R^2^Y 0.934; Q²Y 0.525; P = 0.04. (**D**) OPLS-DA loading plot of SHAM-CT vs SHAM-SR. Negative p(corr) values represent decreased and positive p(corr) values represent increased metabolite concentrations in sleep restriction (SR) compared to control. (**E**) OPLS-DA scores plot for SHAM-CT vs ROT-SR (dark blue, SHAM-CT, n = 11; dark red, ROT-SR, n = 11); R²X 0.499; R²Y 0.920; Q²Y 0.566; P = 0.03. (**F**) OPLS-DA loading plot of SHAM-CT vs ROT-SR. Negative p(corr) values represent decreased and positive p(corr) values represent increased metabolite concentrations in rotenone and SR compared to sham control.

Targeted LC/MS-based metabolic profiling was used to examine the effect of phenotype (ROT vs SHAM) and sleep condition (CT vs SR) on plasma metabolite concentrations. A pair-wise orthogonal partial least-squares discriminant analysis (OPLS-DA) model was obtained comparing the plasma metabolic profiles of SHAM-CT animals with ROT-CT animals (Fig. 3A); R²X = 0.636; R²Y = 0.996; Q²Y = 0.312; n = 1 + 5 components. This model was not significant (CV ANOVA P > 0.05). The corresponding p(corr) loading plot is shown in Fig. 3B and the p(corr) values for each metabolite are presented in Supplementary Table S1A. ROT treatment was associated with increased plasma branched-chain amino acids (BCAA) (leucine (LEU), isoleucine (ILE), valine (VAL)), ornithine and 33 phospholipids (p(corr) > 0.3) and decreased circulating alpha-aminoadipic acid (α-AAA) and propionylcarnitine (AC-C3) (p(corr) < −0.3) (Fig. 3B; Table S1A). By contrast a significant difference was observed in the SHAM-SR group compared to SHAM-CT. A significant pair-wise OPLS-DA model comparing SHAM-CT with SHAM-SR (R²X = 0.421; R²Y = 0.934; Q²Y = 0.525; n = 1 + 2 components; CV ANOVA P = 0.04; validated by permutation analysis) is shown in Fig. 3C, the p(corr) loading plot is shown in Fig. 3D and the p(corr) values for each metabolite are presented in Supplementary Table S1B. Sleep restriction in control animals increased plasma concentrations of the BCAAs (LEU, ILE, VAL), ornithine, arginine, lysine, alanine, proline, phenylalanine (PHE), carnitine (AC-C0), and phospholipids (n = 15) (p(corr) > 0.3) and decreased creatinine, putrescine, symmetric dimethylarginine, kynurenine (KYN), α-AAA, trans-4-hydroxyproline, acetylcarnitine (AC-C2), tetradecenoylcarnitine (AC-C14:1) and glutaconylcarnitine (AC-C5:1-DC) (p(corr) < −0.3) compared to controls. A significant pair-wise comparison OPLS-DA model was also obtained comparing SHAM-CT with ROT-SR (R²X = 0.499; R²Y = 0.920; Q²Y = 0.566; n = 1 + 2 components; CV ANOVA P = 0.03; validated by permutation analysis; Fig. 3E). The p(corr) loading plot is shown in Fig. 3F and the p(corr) values of the metabolites are presented in Supplementary Table S1C. Sleep restriction with ROT increased plasma concentrations of the BCAAs (LEU, ILE, VAL), ornithine, arginine, lysine, proline, PHE, serine, spermidine, the sphingolipid (SM C16:1) and many phospholipids (n = 54) (p(corr) > 0.3) and decreased creatinine, trans-4-hydroxyproline, acetylcarnitine (AC-C2) and tetradecenoylcarnitine (AC-C14:1) (p(corr) < −0.3) compared to controls (SHAM-CT). After rebound, the effects of SR and ROT were lost (SHAM-CT vs ROT-REB) (OPLS-DA model R^2^X = 0.350, R^2^Y = 0.348, Q^2^Y = 0.216; n = 1 + 0 components; CV ANOVA P = 0.113).

**Figure 4:**
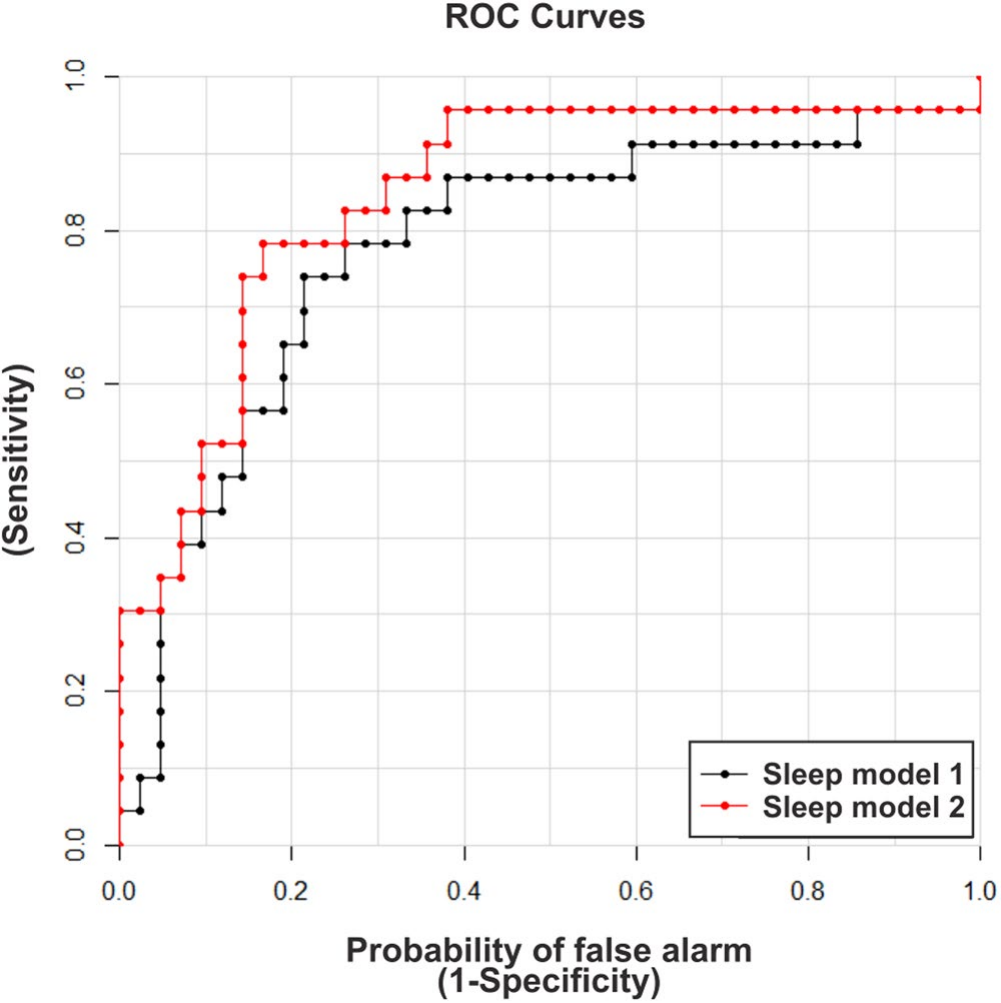
Receiver operating characteristic (ROC) plots of SR prediction models. Models were improved stepwise by including an additional metabolite until no more gain in AUC could be obtained during cross-validation. Model 1: kynurenine (KYN) (black); AUC = 0.777. Model 2: kynurenine and isoleucine (ILE) (red); AUC = 0.845.

ANOVA analysis was performed to assess differences in metabolite concentration ratios. Before this analysis, a heat map combined with hierarchical clustering was performed (Fig. S4A). Statistical analysis with one-way ANOVA (simple six-group - SHAM/ROT - CT, SR and rebound group- comparison, straightforward approach) identified 10 metabolites that were significantly different between the study groups when compared to SHAM-CT (FDR < 0.05): ILE, LEU, VAL, alpha-AAA, KYN, PC aa C30:0, PC aa C34:1, PC aa C40:2, PC aa C42:4, and PC ae C38:3 (shown in Table 1 with FDR values). Multivariate ANOVA including all groups showed 3 significant metabolites (ILE, LEU – increased; KYN – decreased) due to SR if compared to SHAM-CT and no differences for the rotenone lesion. Table 1 shows the metabolite ratios compared to control (SHAM-CT) and FDR values per comparison. Models including interactions did not present any metabolites with FDR < 0.10. Results after rebound show different metabolites altered, either if compared to SR or CT. Supplementary Fig. S4B shows the hits from one-way and multivariate ANOVA combined for all groups, including rebound. Using logistic prediction modeling, models were built to predict whether samples were exposed to ROT, SR or rebound. Starting from no model, models were developed by adding one metabolite at a time to an existing model until no further improvement in AUC was obtained. For ROT, since no metabolites were significant, the best model was only with PC aa C40:2 (FDR = 0.072; AUC = 0.669), adding other metabolites did not improve AUC. For SR, the simplest model was based on KYN and stepwise improvement combined with leave-one-out cross-validation resulted in a final model with KYN and ILE (AUC = 0.845) (Fig. 4A). This model allowed 80% accuracy, 70% sensitivity and 86% specificity. ROT animals were more often correctly predicted as SR (ROT-SR n = 11; n = 9 predicted as SR) than SHAM (SHAM-SR n = 12; n = 7 predicted as SR).

**Table 1:** Metabolite concentration ratios compared to control (SHAM-CT) and FDR values per comparison.

Ratios to CT	SHAM CT	SHAM SR	ROT CT	ROT SR	FDR		
					one-way ANOVA	Multivariate ANOVA	
						SR	Rotenone
Isoleucine	1.00	1.22	1.12	1.35	0.021	**0**.**039**	0.300
Leucine	1.00	1.26	1.18	1.40	0.021	**0**.**039**	0.261
Valine	1.00	1.21	1.12	1.33	0.014	0.061	0.285
Alpha-AAA	1.00	0.87	0.72	0.92	0.032	0.432	0.862
Kynurenine	1.00	0.79	1.15	0.72	0.028	**0**.**014**	0.799
PCaaC30:0	1.00	1.19	1.17	1.36	0.021	0.622	0.275
PCaaC34:1	1.00	1.21	1.17	1.43	0.049	0.529	0.275
PCaaC40:2	1.00	1.02	1.13	1.48	0.028	0.771	0.072
PCaaC42:4	1.00	1.01	1.00	1.26	0.028	0.871	0.489
PCaaC38:3	1.00	1.10	1.27	1.49	0.034	0.357	0.128

Statistical analysis with one-way ANOVA showed 10 metabolites with significant differences (FDR < 0.05) listed here. Multivariate ANOVA showed 3 metabolites significantly altered (highlighted in bold) due to SR in comparison to SHAM-CT and no differences for rotenone lesion.

**Figure 5:**
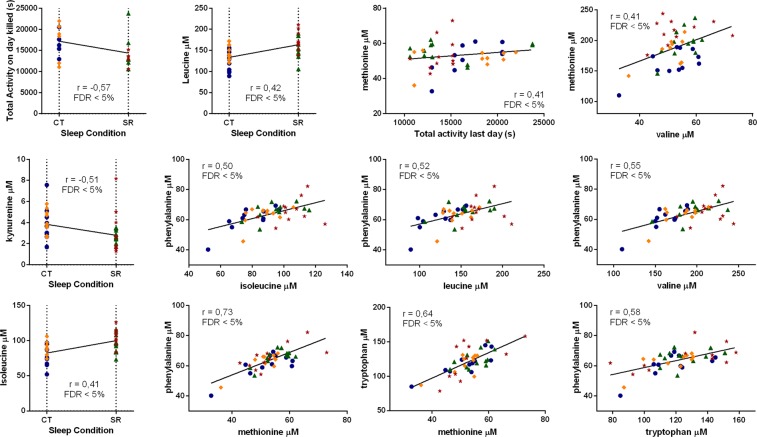
Behavioural and neurochemical correlations. Correlations with an r > 0.4 or <−0.4 were considered since this value is approximately the threshold where the tested correlations become significant with FDR < 0.05. From the top left to the bottom right: total activity on day killed × Sleep Condition (r = −0.57); LEU × Sleep Condition (r = 0.42); total activity on day killed × MET (r = 0.41); MET × VAL (r = 0.41); KYN × Sleep Condition (r = −0.51); PHE × ILE (r = 0.50); PHE × LEU (r = 0.52); PHE × VAL (r = 0.55); ILE × Sleep Condition (r = 0.41); PHE × MET (r = 0.73); TRP × MET (r = 0.64); PHE × TRP (r = 0.58). Colour code: dark blue, SHAM-CT, n = 11; green, SHAM-SR, n = 12; orange, ROT-CT, n = 10; dark red, ROT-SR, n = 11.

We also performed a correlations analysis (Fig. 5) to see if any of the parameters could be associated, considering only the correlation with an r > 0.4 or < −0.4, since this value is approximately the threshold where the tested correlations become significant with FDR < 0.05. For illustration purposes, we show the correlation between sleep condition and total activity on day killed, with r = −0.57. For metabolite concentrations, there was a correlation between SR and KYN (r = −0.51), ILE (r = 0.41) and LEU (r = 0.42). Considering the behavioural and metabolomics data, a correlation between total activity on day killed and methionine (MET) concentrations was demonstrated (r = 0.41). All the other correlations found were between metabolite concentrations, all increased in the model: LEU × ILE (r = 0.90); PHE × ILE (r = 0.50); VAL × ILE (r = 0.94); PHE × LEU (r = 0.52); VAL × LEU (r = 0.89); PHE × MET (r = 0.73); tryptophan (TRP) × MET (r = 0.64); MET × VAL (r = 0.41); PHE × VAL (r = 0.55); PHE × TRP (r = 0.58).

**Figure 6:**
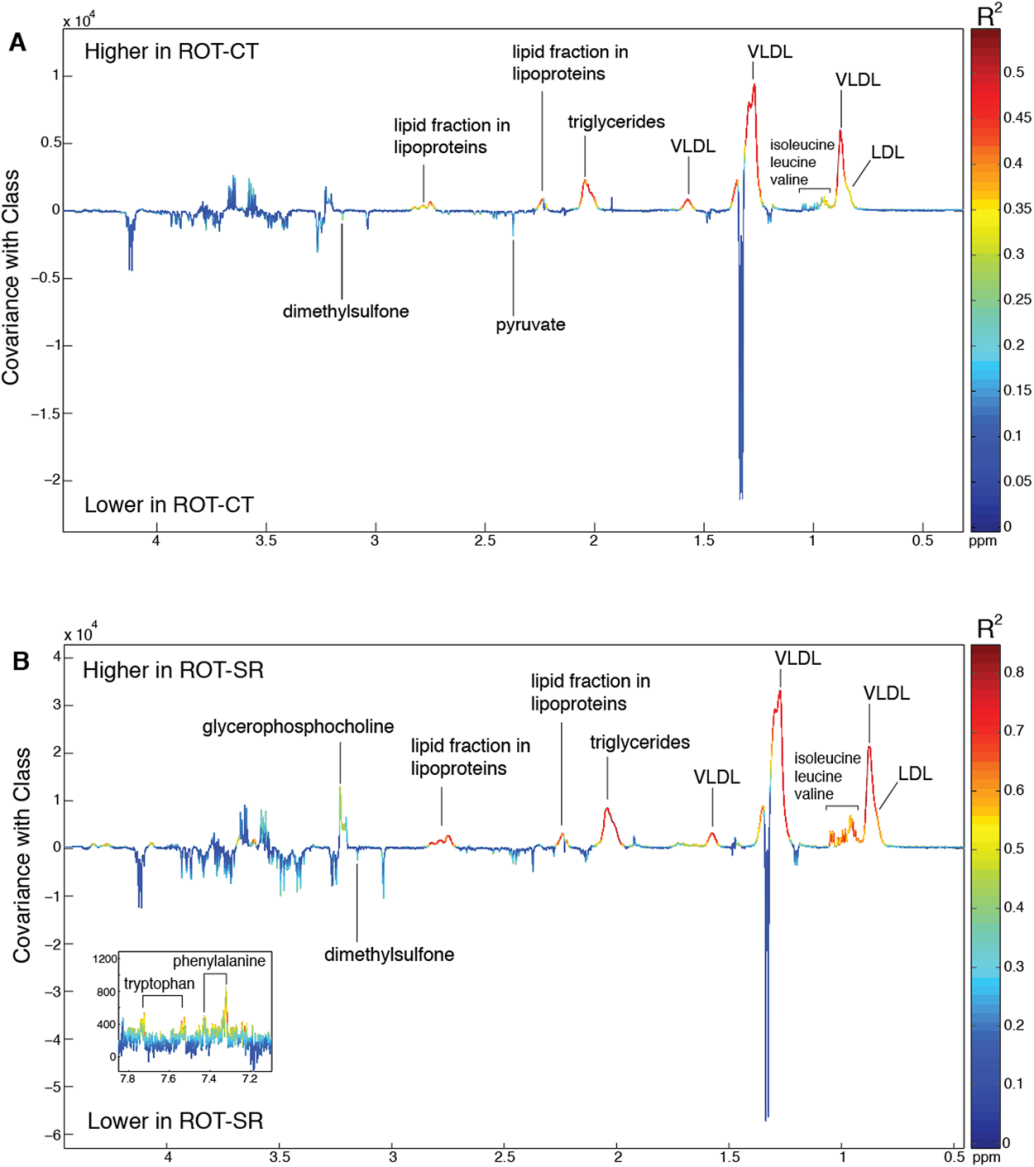
OPLS-DA models comparing the plasma ¹H NMR spectral profiles. (**A**) OPLS-DA from SHAM-CT versus ROT-CT (Q^2^Y = 0.35, P = 0.027). LDL = Low density lipoproteins; VLDL = very low density lipoproteins (dark blue, SHAM-CT, n = 10; orange, ROT-CT, n = 9; dark red, ROT-SR, n = 6). (**B**) OPLS-DA from SHAM-CT versus ROT-SR (Q^2^Y = 0.70, P = 0.001).

¹H NMR spectroscopy was used for untargeted metabolic profiling of the plasma samples. A significant OPLS-DA model was obtained comparing the plasma metabolic profiles between SHAM-CT and ROT-CT groups (R^2^X = 0.56, R^2^Y = 0.75, Q²Y = 0.35*;* P = 0.027 following 999 permutations). The corresponding coefficient plot is shown in Fig. 6A. Plasma from the ROT-CT group contained lower amounts of dimethylsulfone and pyruvate compared to the SHAM-CT group, but the most evident differences were associated with higher circulating amounts of triglycerides and lipoproteins (specifically, low density lipoproteins (LDL) and very-low density lipoproteins (VLDL) in the ROT-CT group). Consistent with the targeted LC/MS metabolomics, there was an increase in all BCAAs in the ROT-CT compared to the SHAM-CT group. However, a comparison between the sham groups (SHAM-CT vs SHAM-SR) did not reveal any significant difference produced by sleep restriction itself, in contrast to LC/MS analysis. Nevertheless, when comparing the additive effects of ROT and SR (ROT-SR) with SHAM-CT, the predictive ability of the OPLS-DA model was stronger (R^2^X = 0.59, R^2^Y = 0.91, Q^2^Y = 0.70, P = 0.001 following 999 permutations; Fig. 6B) than ROT alone (SHAM-CT vs ROT-CT; Q^2^Y = 0.35). While the increase in lipoproteins observed in the ROT-CT group remained, the increase in BCAAs was more pronounced with additional increases in TRP, PHE and glycerophosphocholine (GPC). After rebound, the additive effects produced by SR in ROT were lost (SHAM-CT vs ROT-REB) (OPLS-DA model R^2^X = 0.27, R^2^Y = 0.40, Q^2^Y = 0.19; P = 0.068).

## Discussion

Using the established ROT rodent model of PD, we have shown that chronic SR can affect the behavioural and metabolic characteristics in this model consistent with PD. Collectively, these data reinforce the concept that SR in PD contributes to the development of metabolic shifts early in the disease (equivalent to Braak stages 2–3) that could aggravate its progression and be established as reliable early-phase predictive biomarkers. In addition, our work contributes to the consolidation of the ROT-PD model as a reliable model to study PD features. The ROT-PD has established itself more and more as a prototypical model to study sleep aspects related to the SNpc dopaminergic lesion. Previous studies have shown that both chronic systemic administration and stereotaxic injection of ROT were associated with sleep alterations, as long-term and progressive deterioration of spontaneous sleep^35^ and decrease of the time in NREM sleep^33^.

In the ROT-treated compared to the SHAM animals a selective reduction in the percentage of TH-ir neurons within the SNpc, which is related to several NMS that are landmarks of early-phase features of PD, was observed^9,32,33,37^. Dopamine depletion within the SNpc leads to an increase in striatal D2 receptors, increasing activity of the nigrostriatal indirect-pathway resulting in movement impairment^47^. In addition, a dopaminergic lesion selectively decreases the synaptic strength of thalamic inputs into the direct-pathway, suggesting that motor impairments occur as a result of an imbalanced activation of basal ganglia circuitry by the thalamus, which can be reversed with thalamic inhibition^48^. The reduction in motor activity seen in the ROT-CT group, also observed in other ROT-PD models^39,49^, was reversed in the ROT-SR group, indicating that SR triggers a dopaminergic compensatory mechanism. This is in agreement with Ramanathan, *et al*.^50^, who showed that a 6 h SR protocol of gentle handling produced an increase in exploratory behaviour. Similarly, 24 h of total sleep deprivation produced the same outcome, without changes in plasma corticosterone, suggesting that the increase in locomotor activity was not a consequence of the hypothalamic-pituitary-adrenal stress response^51^. REM sleep deprivation is also able to augment locomotion^39^ and counterbalance PD model-induced hypokinesia^52^, a compensatory effect considered to be due to supersensitivity of brain dopamine receptors^53^, specifically, through D2 receptor up-regulation^54^. Therefore, total SR can lead to a substantial loss of REM sleep, enough to compensate for the motor impairment produced by ROT lesion.

Substantial evidence supports the idea that sleep deprivation or SR impairs memory processes^55–59^ as a result of different mechanisms: (i) reduction of thalamic activity^60^; (ii) decline of hippocampal long-term potentiation^61^; (iii) decrease of hippocampal AMPA receptor function^62^; (iv) brain inflammation and increasing susceptibility to toxins^63^. Therefore, our ORT findings, showing that the ROT-SR group was not able to remember the familiar object, are aligned with the results of Dos Santos, *et al*.^39^, showing that ROT can impair object recognition, but, unlikely the acute REM sleep deprivation which improved memory, our chronic protocol suggest a significant synergistic effect of SR and the dopaminergic ROT lesion in the maintenance of cognitive impairment. It is noteworthy that ORT is a familiarity-based memory task, which is correlated to human episodic-like memory and spatial learning, both impaired in early-stage PD, suggesting that the basal ganglia interact with the prefrontal cortex to mediate high-level cognition^64,65^. Basal ganglia influence cortical learning-dependent synaptic plasticity by selectively modulating corticostriatal inputs; in a dopamine depletion background, this plasticity is altered due to unsuitable filtering by the basal ganglia, resulting in abnormal learning^66^. It is known that significant areas for object recognition in rats are cortical association areas, the mediodorsal thalamic nucleus, the rhinal cortices, especially the perirhinal, and the prefrontal cortical areas^67,68^. These structures have primary projections to the striatum and there is evidence that the striatum modulates information flow to the prefrontal cortex^69^, providing the background for cognitive impairment.

There is also evidence for effects of circadian disruption in long-term object recognition, independent of sleep^70^. The observed rest-activity patterns revealed that the ROT-SR group showed significantly reduced arousal during the first hour of night-time activity (their active phase), suggesting attenuated behavioural arousal and an association between sleep and circadian changes in a PD context. Circadian disruption in PD could reflect not only sleep disturbances but also autonomic, cognitive, psychiatric and motor impairments, further endorsed by the role that dopamine has in sleep regulation and circadian homeostasis^5,7,71^. Lastly, it is plausible that the mechanisms that affect the circadian system in PD could influence sleep/wake processing. Therefore, the occurrence of one reinforces the progression of the other, potentially involving a feedback mechanism^72^, predisposing the brain to neurodegeneration through a number of key mechanisms^73^.

To elucidate the biochemical processes through which chronic SR can enhance the PD phenotype in the ROT model, we combined behavioural testing with targeted LC/MS and untargeted NMR spectroscopy based metabolic profiling. Targeted metabolomics quantifies a large number of known metabolites, enabling the detailed characterization of changes in specific metabolic pathways in response to stimuli (ROT and/or SR)^44^. Untargeted ^1^H NMR spectroscopy-based metabolic profiling is complementary to the targeted approach, measuring an extensive range of metabolites, both endogenous and exogenous, providing a holistic overview of the metabolic system and how it is modulated by different exposures^46^. This dual approach provides wide metabolome coverage and, in this study, significant metabolic alterations were observed in the animals, mainly due to SR associated with the ROT-induced dopaminergic lesion.

ROT treatment was observed to increase the plasma lipoproteins, LDL and VLDL. This occurred both in the presence and absence of SR. VLDL is responsible for transporting energy-rich triacylglycerol to cells in the body while LDL is responsible for delivering cholesterol. Hypercholesterolemia has become a prognostic risk factor for neurodegenerative diseases, even contributing to PD onset^74^. Indeed, elevated cholesterol is associated with the loss of dopaminergic neurons in the SNpc, followed by a reduction in striatal dopamine levels, as a product of mitochondrial dysfunction, that leads to motor impairment^75^. Lipoprotein lipase (LPL) is the enzyme that removes triacylglycerol from VLDL transforming it into LDL. LPL is highly expressed in hippocampal neurons. Additionally, LPL is involved in the pathogenesis of dementia^76–78^ and LPL-deficient mice have been shown to have presynaptic dysfunction and impaired memory function^79^. Interestingly, ROT lesion was already linked to lipid impairment, with alterations in polyunsaturated fatty acid cardiolipin in the SNpc and plasma in rats^80^.

A family of metabolites suggested to be involved in lipid metabolism, implicated in insulin resistance and obesity, are the BCAAs^81^, which were found to be significantly elevated in our model. In SR rats compared to non-SR controls, LEU, ILE and KYN were significantly altered, with a statistical correlation for the sleep condition. Although LEU and ILE were increased in all groups in comparison with SHAM-CT, the most prominent increment was in the ROT-SR group. The remaining BCAA, VAL, showed a non-significant trend (P = 0.061) to increase (measured by targeted metabolomics), which was corroborated by the untargeted approach with all BCAAs showing increased plasma levels in the ROT-SR condition (there was also a slight increase in ROT-CT only, but SR appears to enhance this effect). These findings are in accordance with previous studies that have shown increased BCAA levels in PD patients, notably ILE and LEU^82,83^, and in PD animal models^84^. The pathophysiological significance of these changes is still unclear, but it is known that impaired BCAA metabolism results in greater circulation of these BCAAs, and consequent mitochondrial dysfunction^85^. They are also transported into the brain via the large neutral amino acid transporter, which also transports TRP, PHE and tyrosine (TYR). Increased plasma BCAA levels reduce TRP and TYR transport into the brain due to competition for the carrier at the blood-brain barrier, influencing synthesis and release of serotonin and catecholamines (most notably dopamine, by reducing tyrosine available for hydroxylation) within the brain^86^. TRP is the precursor of serotonin, which is involved in sleep/wake regulation, functioning primarily to promote wakefulness and closely related to memory and emotional processes in PD^37,87^. Competition between BCAA and TRP/TYR for brain uptake could, therefore, negatively impact serotonin and dopamine metabolism, contributing to the occurrence of NMS in PD.

Interestingly, TRP was also found to be higher in the plasma of the ROT-SR group compared to the SHAM-CT group (measured by NMR). TRP metabolism was found to be altered in early-stage PD patients in the study of Luan, *et al*.^82^, with TRP catabolites increased in the urine compared to healthy subjects, particularly KYN. There are some studies showing the involvement of KYN metabolism in PD^88–90^, and in our plasma samples, KYN was found to be decreased as a result of SR but not ROT. The KYN pathway, after its conversion from TRP by indoleamine 2,3-dioxygenase, produces three metabolites that have important roles in the brain: two of them, quinolinic acid and 3-hydroxyl-kynurenine, are NMDA receptor agonists and have neurotoxic effects, producing progressive mitochondrial dysfunction; the other one, kynurenic acid (KYNA), has a neuroprotective effect, acting as a free radical scavenger and glutamate antagonist^91^. In mammals, about 40% of KYN is produced in the brain, whereas 60% of it is taken up from the periphery^92^. Therefore, if there is a transport impairment of TRP in the blood-brain barrier due to competition with BCAA, it could reflect in an increased uptake of KYN from the periphery. On the other hand, TRP pathway could be shifted towards serotonin synthesis, since, as previously mentioned, it is known to promote wakefulness^87^. Beyond that, LEU, ILE, MET and PHE have been shown to reduce tissue KYN concentrations and suppress KYNA synthesis in a dose-dependent manner^93^. Since these metabolites were found to be significantly increased in the SR group and even more increased in the ROT-SR group, they could be modulating KYNA formation and therefore KYN metabolism. In addition, using logistic regression prediction, a model with 80% accuracy, 70% sensitivity and 86% specificity was obtained for SR using KYN and ILE (i.e. 70% of SR animals were predicted correctly based on their plasma KYN and ILE abundance), corroborating a relationship between both metabolites.

The other amino acids that were altered in our PD model were PHE and MET, with a positive correlation with BCAA and TRP levels in the LC/MS analysis; likewise, PHE was increased in the ROT-SR group in the untargeted NMR analysis. PHE is the precursor of DA, reported to be altered in PD^82,94^, and has been associated with alterations in sleep patterns^95^. MET levels on day killed had a positive correlation with the total activity on the last day. The administration of MET, an acetylcholine precursor, was found to increase diurnal activity in birds, an effect supposed to be due acetylcholine stimulation^96^; MET is also implicated in PD, mainly through its metabolite homocysteine, since elevated plasma levels of homocysteine have been observed in PD^97^. The observed increase in ornithine and arginine levels could be indicative of impairment in the urea cycle; ornithine has previously been reported to be increased in PD, purportedly due to a reduction in the ability of mitochondria to transport ornithine for conversion in the urea cycle^83^ and also as a consequence of ROT exposure, which inhibits the activity of ornithine decarboxylase (Rhee *et al*., 2016). Arginine, in turn, is also part of the nitric oxide pathway, which has already been implicated in PD through nitrosative stress^98^, reported to be higher in PD patients^99^, and in the initial phase of PD animal models, being inversely correlated with TH immunolabeling^100^.

ROT exposure in combination with sleep deprivation also resulted in an increase in plasma GPC. GPC, a cholinergic precursor, is one of the major phosphorus containing-choline components^101^, and is a precursor of acetylcholine, a neurotransmitter necessary for normal cognitive function, including learning and memory^102^. In PD patients with mild cognitive impairment, brain GPC abundance has also been found to be increased^103^, although it was decreased in cerebrospinal fluid in idiopathic PD^104^. In addition, thalamic GPC spectroscopy analysis has been shown to be useful in differentiating tremor-dominant PD patients from those with resting tremor in essential tremor^105^. In the SR context, it has previously been reported that GPC concentrations were increased following recovery from a night of sleep deprivation^101^. It is known that choline transport through the blood-brain barrier decreases with age contributing to degenerative processes^106^; therefore, the findings observed here could be related to a reduction in choline transport, with a consequent increase in plasma GPC levels.

Although one can arguably question the validity of the rotenone model, in fact our work contributes to understand the underlying pathophysiology of the SNpc dopaminergic neurodegeneration. Still, some limitations of the study should be highlighted: sleep measurement was limited to behavioural rest-activity analysis and lacked a physiological measure of sleep (i.e., EEG) to determine the impact of the sleep restriction on the sleep architecture; the absence of quantification of proteins, for example, indoleamine 2,3-dioxygenase, quinolinic acid and 3-hydroxyl-kynurenine, from TRP and KYN pathway, or lipoprotein lipase, to correlate with the lipid alterations, in order to better understand the mechanism underlying the observed alterations; failure to establish the extent of the lesion in each animal to compare with the variation in the measured parameters. Nevertheless, these limitations open a research agenda to keep investigating these findings and fully understand the role of these metabolites and metabolic pathways in PD pathophysiology.

In conclusion, in our model, we have observed that chronic SR produces behavioural and metabolic alterations that reveal for the first time to our knowledge a novel set of metabolic pathways, which are involved in PD pathophysiology. Sleep restriction in the early-phase of PD can exacerbate and modulate the biochemical changes associated with disease progression. These metabolic alterations could represent reliable and sensitive markers of early-phase PD and may help track progression of the disease before the appearance of motor signs. Identification of such individuals has the potential to improve therapeutic strategies and possibly delay or attenuate the onset of symptoms which amplify the behavioural and metabolic characteristics in this model consistent with PD. Collectively, our findings reinforce the concept that SR in PD contributes to the development of metabolic shifts early in the disease (parallel to the non-motor phase) that could aggravate its progression and be established as reliable early-phase predictive biomarkers.

## Supplementary information

Supplementary Information

## Data Availability

The datasets used and/or analysed during the current study are included in this published article [and its supplementary information files]. If any additional information is required, it is available from the corresponding author on request.
