# Supplementary material for: Chronic sleep restriction in the rotenone Parkinson’s disease model in rats reveals peripheral early-phase biomarkers
Source: Sci Rep. 2019 Feb 13;9:1898. doi: 10.1038/s41598-018-37657-6 (PMC6374389; doi:10.1038/s41598-018-37657-6)
Supplement: Supplementary file 1 — Supplementary Information [file 41598_2018_37657_MOESM1_ESM.docx]

**Chronic sleep restriction in the rotenone Parkinson’s disease model in rats reveals peripheral early-phase biomarkers**

Juliane Fagotti^1^, Adriano D S Targa^1^, Lais S Rodrigues^1^, Ana Carolina D Noseda^1^, Flávia W C Dorieux^1^, Franciele F Scarante^1^, Jessica L Ilkiw^1^, Fernando M Louzada^1^, Namrata R Chowdhury^2^, Daan R van der Veen^2^, Benita Middleton^2^, Jeroen L A Pennings^3^, Jonathan R Swann^4^, Debra J Skene^2^ and Marcelo M S Lima^1*^

**Supplementary Information**

**SI Figures and Tables**

**
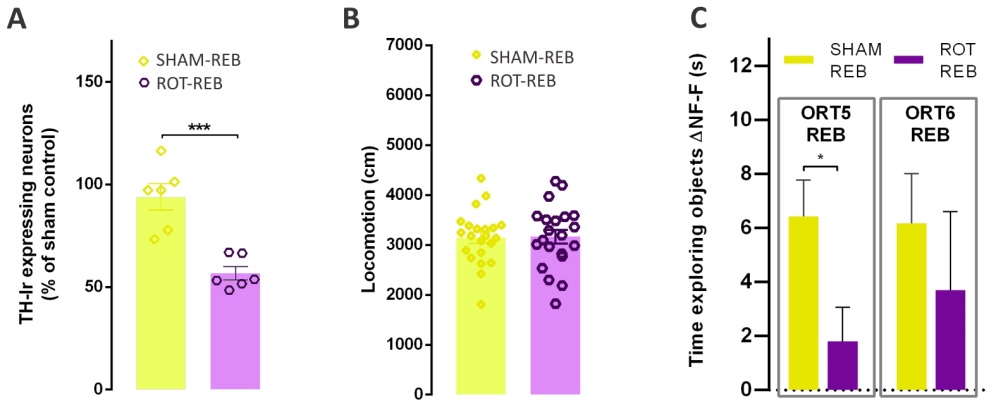
**

**Figure S1. Immunohistochemistry and behavioural parameters after rebound (REB).**

(A) Percentage of TH-ir expressing neurons in the SNpc in relation to the SHAM-CT group. Individual data are shown as a scatter plot with bar of mean ± standard error of the mean (SEM n = 6 per group. ***P ≤ 0.001 (Unpaired t-test).

(B) Locomotion parameter obtained from the open field test. Individual data (cm) are shown as a scatter plot with bar of mean ± SEM. Unpaired t-test shows no significant differences. n = 22 SHAM-REB; n = 21 ROT-REB.

(C) Exploration index (ΔNF – F). The bars represent the mean time exploring objects (s) ± SEM one (ORT5) and two weeks (ORT6) after sleep restriction. *P < 0.05 (two-way ANOVA followed by Fisher’s LSD test). n = 22 SHAM; n = 21 ROT. NF = non-familiar object; F = familiar object.

**
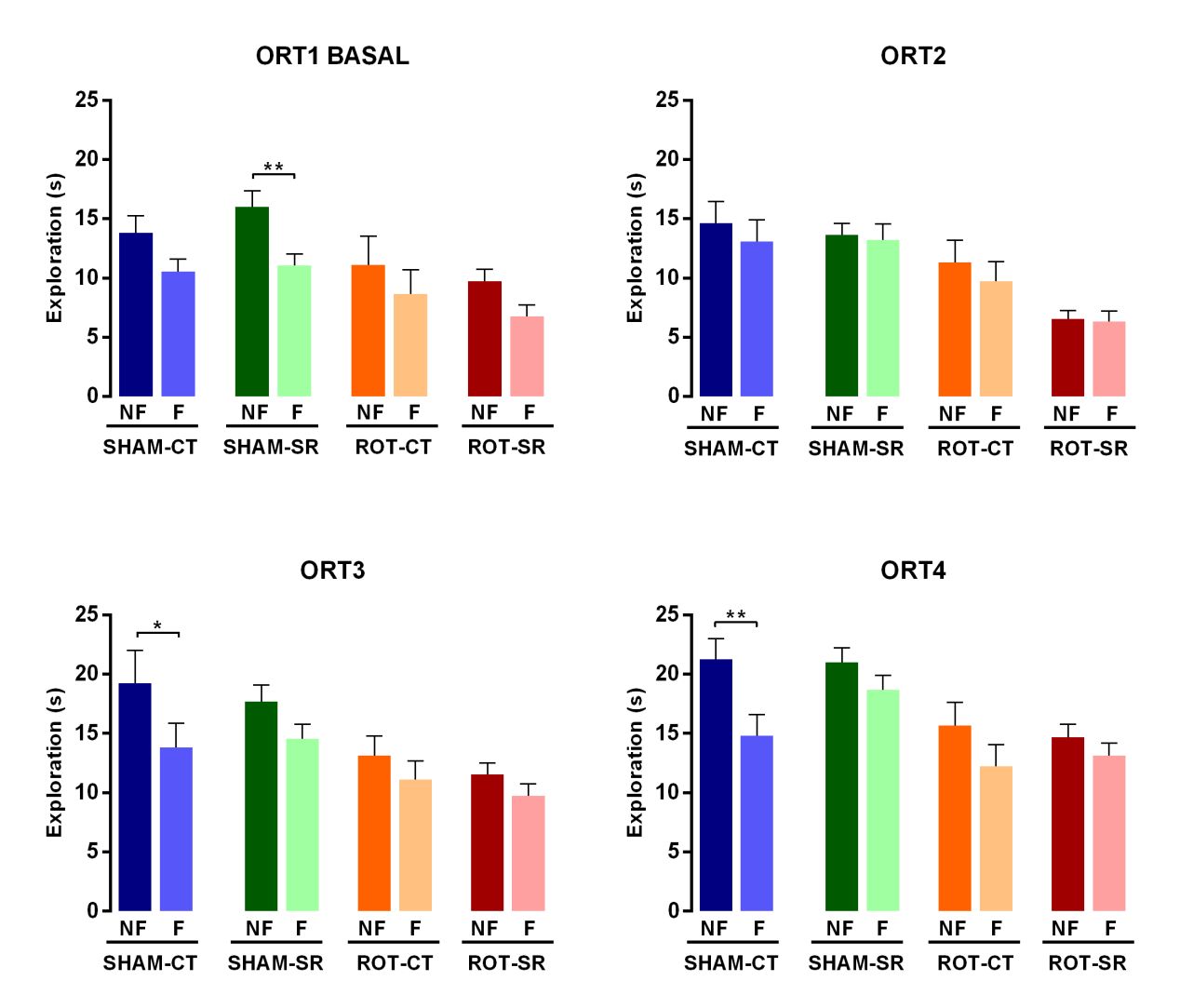
**

**Figure S2. Duration of object exploration in Object Recognition Task.**

The bars represent the mean ± SEM exploration time (s) before and during 3 weeks of sleep restriction (SR). *P < 0.05; **P ≤ 0.01 (two-way ANOVA followed by Fisher’s LSD test). n = 24 SHAM-CT; n = 24 SHAM-SR; n = 20 ROT-CT; n = 20 ROT-SR. NF: Non-familiar object; F: familiar object.


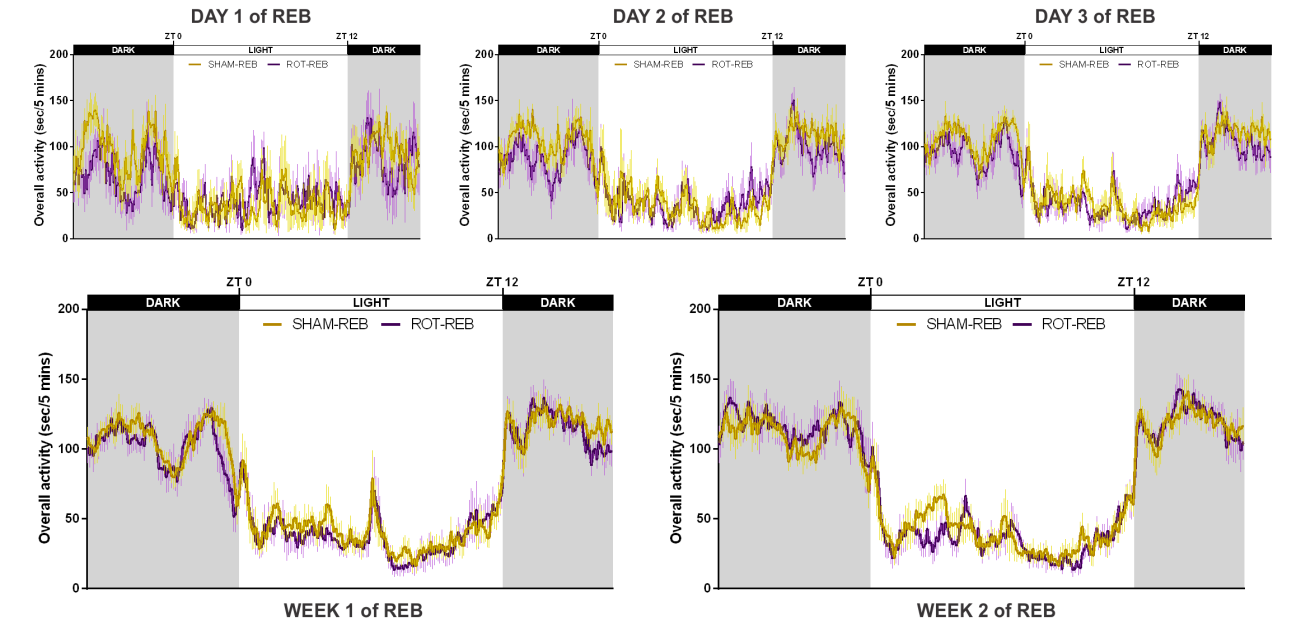


**Figure S3. Rest-activity parameters.**

(A) Daily activity pattern of each group (SHAM-REB and ROT-REB) during sleep rebound period (REB). Each line represents the mean activity (seconds/5 minutes) of the 21 days ± SEM. ZT0 = 07.00 h - lights on, n = 6 per group.


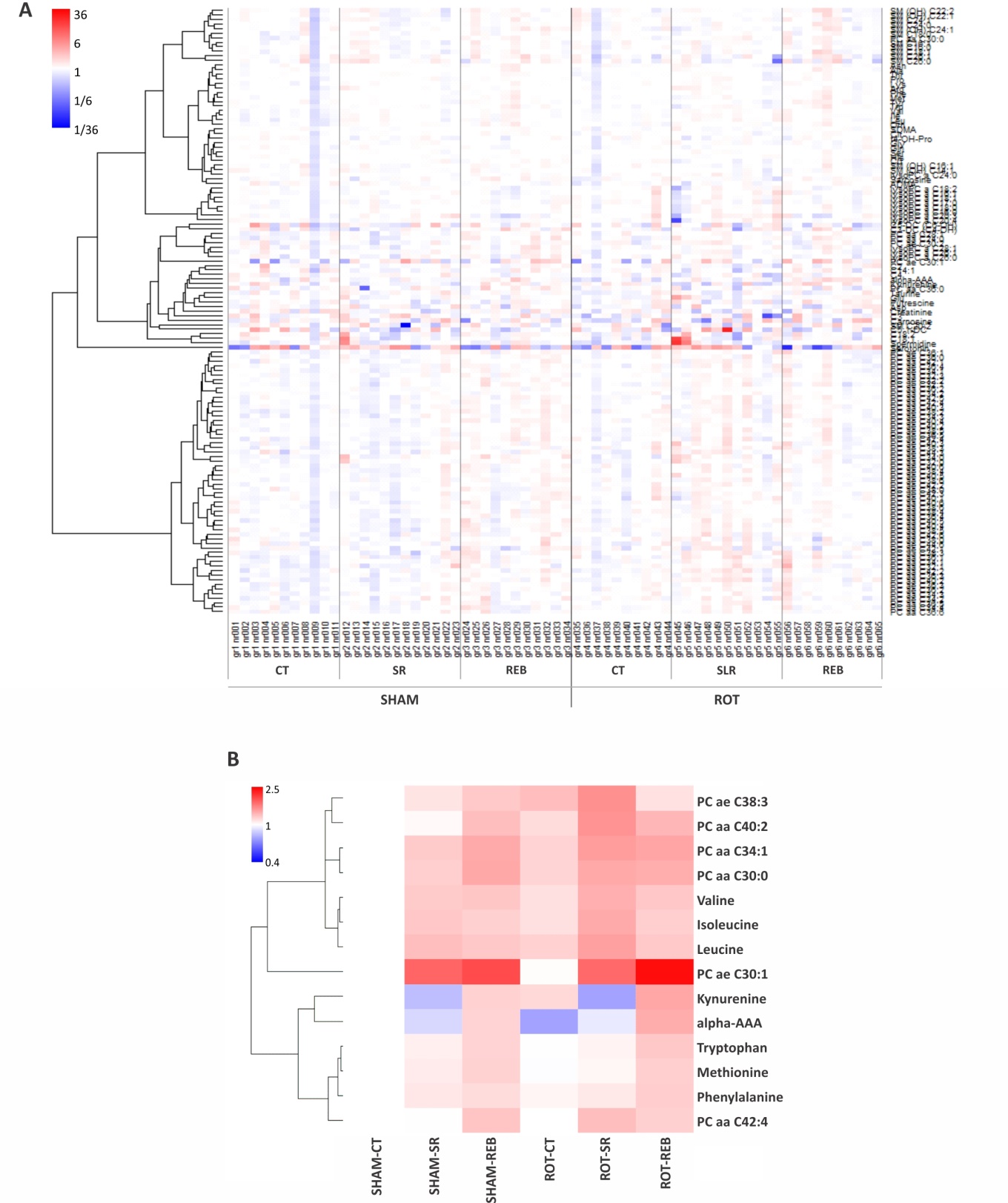


**Figure S4. Multivariate analysis of LC/MS metabolomics data.**

(A) Heat map combined with hierarchical clustering (Euclidean distance and Ward linkage) of metabolites and experimental groups (n = 6). Colour values are based on the relative value compared to the average value of each metabolite in the complete data set. Higher levels of metabolites are shown in red, lower levels are shown in blue.

(B) Hits from one-way and multivariate ANOVA of metabolites showing significant changes between the experimental groups. Higher levels of metabolites are shown in red, lower levels are shown in blue.

**Table S1: OPLS-DA loadings (p(corr) values) of the measured metabolites in the different models**

| **A. Phenotype**  **SHAM vs ROT** | |  | **B. Sleep restriction**  **CT vs SR** | |  | **C. Phenotype + sleep restriction** | |
| --- | --- | --- | --- | --- | --- | --- | --- |
| **SHAM-CT vs ROT-CT** | |  | **SHAM-CT vs SHAM-SR** | |  | **SHAM-CT vs ROT-SR** | |
| **Metabolite** | **p(corr)** |  | **Metabolite** | **p(corr)** |  | **Metabolite** | **p(corr)** |
| **alpha-Aminoadipic acid** | -0.415 |  | **Creatinine** | -0.554 |  | **Creatinine** | -0.422 |
| **Propionylcarnitine** | -0.336 |  | **Putrescine** | -0.530 |  | **trans-4-hydroxyproline** | -0.409 |
| **Glutamate** | -0.282 |  | **Tetradecenoylcarnitine** | -0.395 |  | **Acetylcarnitine** | -0.386 |
| **Tyrosine** | -0.279 |  | **Symmetric dimethylarginine** | -0.354 |  | **Tetradecenoylcarnitine** | -0.362 |
| **Asymmetric dimethylarginine** | -0.277 |  | **Kynurenine** | -0.320 |  | **Tyrosine** | -0.277 |
| **Octadecadienylcarnitine** | -0.257 |  | **alpha-Aminoadipic acid** | -0.308 |  | **Asymmetric dimethylarginine** | -0.266 |
| **Octadecanoylcarnitine** | -0.233 |  | **trans-4-hydroxyproline** | -0.301 |  | **Symmetric dimethylarginine** | -0.237 |
| **Spermidine** | -0.224 |  | **Glutaconylcarnitine** | -0.293 |  | **Kynurenine** | -0.235 |
| **Taurine** | -0.221 |  | **Acetylcarnitine** | -0.283 |  | **SM C26:0** | -0.233 |
| **Acetylcarnitine** | -0.209 |  | **Malonylhydroxybutyrylcarnitine** | -0.247 |  | **Propionylcarnitine** | -0.217 |
| **Creatinine** | -0.195 |  | **lysoPC a C28:1** | -0.237 |  | **Carnitine** | -0.206 |
| **PC ae C36:5** | -0.192 |  | **PC ae C42:1** | -0.180 |  | **Butyrylcarnitine** | -0.200 |
| **trans-4-hydroxyproline** | -0.180 |  | **PC ae C36:5** | -0.175 |  | **PC aa C36:0** | -0.196 |
| **PC ae C44:6** | -0.179 |  | **PC ae C30:2** | -0.154 |  | **SM (OH) C14:1** | -0.139 |
| **Symmetric dimethylarginine** | -0.177 |  | **SM (OH) C22:1** | -0.109 |  | **alpha-Aminoadipic acid** | -0.137 |
| **PC aa C36:0** | -0.173 |  | **PC aa C40:4** | -0.095 |  | **SM (OH) C24:1** | -0.124 |
| **PC aa C42:1** | -0.159 |  | **Asymmetric dimethylarginine** | -0.094 |  | **Sarcosine** | -0.112 |
| **Tetradecanoylcarnitine** | -0.157 |  | **PC aa C38:5** | -0.084 |  | **SM (OH) C22:1** | -0.090 |
| **Threonine** | -0.154 |  | **Glutarylcarnitine** | -0.084 |  | **Glutamine** | -0.086 |
| **Malonylhydroxybutyrylcarnitine** | -0.148 |  | **Tyrosine** | -0.061 |  | **Putrescine** | -0.064 |
| **lysoPC a C26:1** | -0.141 |  | **PC aa C38:0** | -0.043 |  | **SM C20:2** | -0.060 |
| **Glutaconylcarnitine** | -0.140 |  | **SM (OH) C14:1** | -0.042 |  | **SM C26:1** | -0.054 |
| **Serotonin** | -0.134 |  | **lysoPC a C24:0** | -0.035 |  | **lysoPC a C20:4** | -0.047 |
| **SM C26:1** | -0.131 |  | **PC aa C36:0** | -0.028 |  | **SM (OH) C16:1** | -0.045 |
| **SM C26:0** | -0.126 |  | **Octadecadienylcarnitine** | -0.026 |  | **Taurine** | -0.026 |
| **Glutarylcarnitine** | -0.109 |  | **PC aa C40:6** | -0.021 |  | **Glutarylcarnitine** | -0.025 |
| **PC ae C32:1** | -0.086 |  | **PC ae C40:4** | -0.011 |  | **Malonylhydroxybutyrylcarnitine** | -0.010 |
| **Butyrylcarnitine** | -0.081 |  | **PC aa C42:1** | -0.008 |  | **Aspartate** | 0.003 |
| **PC aa C42:5** | -0.080 |  | **PC ae C44:6** | 0.001 |  | **Citrulline** | 0.042 |
| **SM (OH) C14:1** | -0.066 |  | **lysoPC a C26:1** | 0.002 |  | **Octadecadienylcarnitine** | 0.046 |
| **Carnosine** | -0.060 |  | **PC ae C38:0** | 0.003 |  | **lysoPC a C28:1** | 0.060 |
| **Methionine** | -0.059 |  | **PC aa C38:4** | 0.008 |  | **PC ae C44:6** | 0.070 |
| **Alanine** | -0.053 |  | **SM C16:0** | 0.016 |  | **lysoPC a C18:2** | 0.087 |
| **PC aa C38:0** | -0.052 |  | **PC ae C44:3** | 0.021 |  | **lysoPC a C26:1** | 0.091 |
| **Glycine** | -0.052 |  | **SM C20:2** | 0.025 |  | **Glycine** | 0.097 |
| **Tryptophan** | -0.050 |  | **SM C24:0** | 0.040 |  | **Serotonin** | 0.097 |
| **PC ae C30:2** | -0.048 |  | **SM C18:1** | 0.050 |  | **SM (OH) C22:2** | 0.098 |
| **Putrescine** | -0.029 |  | **PC ae C42:4** | 0.050 |  | **Carnosine** | 0.108 |
| **PC ae C44:3** | -0.026 |  | **PC aa C42:5** | 0.065 |  | **SM C24:1** | 0.109 |
| **Aspartate** | -0.010 |  | **lysoPC a C26:0** | 0.070 |  | **SM C16:0** | 0.121 |
| **SM C18:1** | -0.001 |  | **PC aa C34:2** | 0.073 |  | **PC ae C42:1** | 0.128 |
| **PC aa C42:4** | 0.002 |  | **PC aa C32:3** | 0.073 |  | **lysoPC a C18:1** | 0.142 |
| **Serine** | 0.006 |  | **SM (OH) C24:1** | 0.079 |  | **Methionine** | 0.144 |
| **SM (OH) C16:1** | 0.012 |  | **SM (OH) C22:2** | 0.084 |  | **SM C24:0** | 0.151 |
| **PC ae C34:0** | 0.015 |  | **PC aa C36:4** | 0.096 |  | **Tryptophan** | 0.153 |
| **SM C16:0** | 0.018 |  | **PC aa C40:3** | 0.110 |  | **Threonine** | 0.197 |
| **SM (OH) C22:2** | 0.020 |  | **PC aa C40:5** | 0.115 |  | **PC ae C36:5** | 0.199 |
| **Glutamine** | 0.020 |  | **PC ae C38:5** | 0.120 |  | **Glutaconylcarnitine** | 0.213 |
| **Sarcosine** | 0.024 |  | **lysoPC a C20:4** | 0.120 |  | **SM C18:1** | 0.217 |
| **PC aa C32:0** | 0.027 |  | **Serotonin** | 0.130 |  | **PC aa C34:2** | 0.218 |
| **lysoPC a C26:0** | 0.029 |  | **Sarcosine** | 0.133 |  | **lysoPC a C24:0** | 0.226 |
| **PC aa C42:6** | 0.038 |  | PC aa C34:4 | 0.134 |  | **PC ae C38:0** | 0.230 |
| **SM (OH) C24:1** | 0.039 |  | **PC ae C40:3** | 0.153 |  | **lysoPC a C17:0** | 0.232 |
| **PC aa C40:4** | 0.043 |  | **SM (OH) C16:1** | 0.160 |  | **Alanine** | 0.237 |
| **lysoPC a C28:1** | 0.055 |  | PC aa C42:4 | 0.160 |  | **Histidine** | 0.245 |
| **Proline** | 0.056 |  | **Glutamine** | 0.162 |  | **PC ae C40:4** | 0.247 |
| **PC ae C32:2** | 0.060 |  | PC aa C40:2 | 0.169 |  | **PC aa C42:1** | 0.248 |
| **Citrulline** | 0.066 |  | PC aa C42:6 | 0.173 |  | **PC ae C42:3** | 0.257 |
| **Asparagine** | 0.070 |  | **Citrulline** | 0.182 |  | **PC aa C38:0** | 0.263 |
| **PC aa C24:0** | 0.074 |  | **PC ae C40:2** | 0.186 |  | **Glutamate** | 0.267 |
| **PC aa C36:6** | 0.076 |  | **PC aa C24:0** | 0.194 |  | **PC aa C38:5** | 0.274 |
| **PC aa C38:5** | 0.091 |  | **PC ae C32:2** | 0.194 |  | **lysoPC a C26:0** | 0.274 |
| **SM (OH) C22:1** | 0.092 |  | **PC ae C40:5** | 0.216 |  | **SM C18:0** | 0.291 |
| **PC ae C42:1** | 0.097 |  | **Octadecanoylcarnitine** | 0.218 |  | **lysoPC a C18:0** | 0.292 |
| **PC ae C42:4** | 0.112 |  | **SM C26:1** | 0.220 |  | **PC ae C32:1** | 0.292 |
| **Histidine** | 0.115 |  | **PC ae C32:1** | 0.221 |  | **Asparagine** | 0.292 |
| **SM C24:0** | 0.116 |  | **PC ae C38:4** | 0.240 |  | **Octadecanoylcarnitine** | 0.296 |
| **SM C20:2** | 0.121 |  | **SM C24:1** | 0.241 |  | **PC aa C32:3** | 0.298 |
| **PC aa C42:2** | 0.125 |  | **Propionylcarnitine** | 0.249 |  | **PC aa C42:6** | 0.305 |
| **lysoPC a C24:0** | 0.125 |  | **PC ae C40:1** | 0.252 |  | **PC aa C38:4** | 0.309 |
| **PC ae C38:5** | 0.129 |  | **PC aa C28:1** | 0.256 |  | **PC ae C38:6** | 0.311 |
| **PC aa C40:6** | 0.130 |  | **Glycine** | 0.258 |  | **PC ae C30:2** | 0.316 |
| **lysoPC a C20:4** | 0.131 |  | **PC aa C36:6** | 0.264 |  | **PC ae C42:4** | 0.318 |
| **PC ae C30:1** | 0.132 |  | **Butyrylcarnitine** | 0.266 |  | **PC ae C32:2** | 0.322 |
| **PC aa C36:5** | 0.133 |  | **Glutamate** | 0.270 |  | **PC ae C40:1** | 0.324 |
| **Kynurenine** | 0.144 |  | **PC aa C36:5** | 0.271 |  | **PC ae C38:5** | 0.324 |
| **PC ae C36:4** | 0.147 |  | **lysoPC a C18:0** | 0.273 |  | **PC aa C36:6** | 0.326 |
| **PC aa C32:3** | 0.149 |  | **PC ae C42:3** | 0.280 |  | **Spermidine** | 0.328 |
| **Phenylalanine** | 0.151 |  | **PC ae C36:4** | 0.281 |  | **Serine** | 0.330 |
| **Arginine** | 0.177 |  | **PC aa C38:3** | 0.282 |  | **PC ae C44:3** | 0.334 |
| **Lysine** | 0.180 |  | **SM C16:1** | 0.286 |  | **lysoPC a C16:0** | 0.336 |
| **PC aa C34:2** | 0.191 |  | **Serine** | 0.290 |  | **Phenylalanine** | 0.337 |
| **PC aa C38:4** | 0.191 |  | **Histidine** | 0.299 |  | **PC aa C42:5** | 0.344 |
| **PC aa C34:4** | 0.192 |  | **lysoPC a C18:1** | 0.311 |  | **Proline** | 0.347 |
| **SM C18:0** | 0.196 |  | **SM C18:0** | 0.329 |  | **lysoPC a C16:1** | 0.353 |
| **PC aa C36:4** | 0.197 |  | **PC ae C40:6** | 0.333 |  | **PC aa C38:6** | 0.361 |
| **PC aa C40:5** | 0.201 |  | **PC aa C34:3** | 0.338 |  | **PC aa C40:4** | 0.396 |
| **PC ae C38:0** | 0.211 |  | **PC aa C36:2** | 0.344 |  | **PC ae C30:1** | 0.401 |
| **SM C16:1** | 0.221 |  | **PC ae C38:2** | 0.344 |  | **PC ae C34:3** | 0.405 |
| **PC ae C40:3** | 0.236 |  | **PC ae C34:3** | 0.353 |  | **PC ae C40:6** | 0.409 |
| **PC aa C28:1** | 0.246 |  | **Tryptophan** | 0.363 |  | **lysoPC a C20:3** | 0.412 |
| **Carnitine** | 0.257 |  | **SM C26:0** | 0.369 |  | **PC ae C36:4** | 0.417 |
| **PC ae C40:4** | 0.277 |  | **PC ae C34:1** | 0.369 |  | **SM C16:1** | 0.419 |
| **PC ae C42:3** | 0.287 |  | **Asparagine** | 0.378 |  | **PC aa C34:4** | 0.421 |
| **PC aa C40:3** | 0.291 |  | **Spermidine** | 0.378 |  | **PC aa C40:3** | 0.424 |
| **SM C24:1** | 0.303 |  | **Taurine** | 0.384 |  | **PC aa C28:1** | 0.425 |
| **lysoPC a C18:2** | 0.308 |  | **PC ae C38:6** | 0.386 |  | **PC aa C40:6** | 0.436 |
| **PC ae C40:1** | 0.310 |  | **Threonine** | 0.392 |  | **Lysine** | 0.463 |
| **PC aa C40:2** | 0.311 |  | **Methionine** | 0.398 |  | **PC ae C36:0** | 0.468 |
| **lysoPC a C18:0** | 0.317 |  | **Carnosine** | 0.400 |  | **PC aa C36:5** | 0.485 |
| **PC ae C34:1** | 0.327 |  | **PC aa C32:1** | 0.406 |  | **PC aa C36:2** | 0.491 |
| **PC ae C34:3** | 0.331 |  | **PC ae C42:2** | 0.409 |  | **PC aa C32:0** | 0.492 |
| **PC aa C38:6** | 0.338 |  | **PC ae C36:0** | 0.414 |  | **PC ae C34:0** | 0.498 |
| **PC ae C36:0** | 0.345 |  | **lysoPC a C16:1** | 0.419 |  | **PC ae C38:2** | 0.501 |
| **PC aa C38:3** | 0.346 |  | **PC aa C32:0** | 0.425 |  | **PC ae C40:2** | 0.503 |
| **Isoleucine** | 0.347 |  | **PC ae C38:3** | 0.430 |  | **PC ae C38:4** | 0.509 |
| **PC ae C38:4** | 0.348 |  | **Aspartate** | 0.432 |  | **PC ae C34:1** | 0.510 |
| **lysoPC a C18:1** | 0.349 |  | **PC aa C42:2** | 0.436 |  | **PC aa C36:4** | 0.514 |
| **Ornithine** | 0.351 |  | **Lysine** | 0.444 |  | **Arginine** | 0.514 |
| **PC ae C40:2** | 0.355 |  | **Alanine** | 0.452 |  | **PC aa C42:4** | 0.521 |
| **lysoPC a C16:1** | 0.365 |  | **PC ae C36:3** | 0.453 |  | **PC ae C34:2** | 0.541 |
| **PC aa C36:1** | 0.367 |  | **lysoPC a C18:2** | 0.463 |  | **PC aa C24:0** | 0.546 |
| **PC ae C42:2** | 0.383 |  | **PC ae C36:1** | 0.467 |  | **PC aa C40:5** | 0.547 |
| **Valine** | 0.396 |  | **lysoPC a C16:0** | 0.471 |  | **PC ae C40:3** | 0.548 |
| **PC aa C30:0** | 0.398 |  | **Phenylalanine** | 0.471 |  | **PC ae C40:5** | 0.565 |
| **lysoPC a C20:3** | 0.402 |  | **PC ae C34:0** | 0.475 |  | **PC aa C42:2** | 0.577 |
| **lysoPC a C16:0** | 0.404 |  | **Carnitine** | 0.480 |  | **PC ae C36:3** | 0.588 |
| **PC ae C40:5** | 0.405 |  | **lysoPC a C17:0** | 0.496 |  | **PC aa C36:3** | 0.598 |
| **PC ae C38:6** | 0.405 |  | **Arginine** | 0.500 |  | **PC aa C34:3** | 0.599 |
| **PC aa C34:1** | 0.406 |  | **PC aa C38:6** | 0.504 |  | **PC ae C42:2** | 0.604 |
| **PC aa C36:2** | 0.413 |  | **PC ae C30:1** | 0.508 |  | **PC ae C36:1** | 0.629 |
| **PC aa C36:3** | 0.415 |  | **PC aa C36:1** | 0.530 |  | **PC aa C36:1** | 0.650 |
| **lysoPC a C17:0** | 0.436 |  | **PC aa C30:0** | 0.541 |  | **PC aa C40:2** | 0.674 |
| **PC aa C34:3** | 0.436 |  | **lysoPC a C20:3** | 0.549 |  | **PC aa C38:3** | 0.674 |
| **PC ae C36:3** | 0.439 |  | **Proline** | 0.549 |  | **PC aa C30:0** | 0.677 |
| **PC ae C40:6** | 0.452 |  | **PC aa C36:3** | 0.557 |  | **Ornithine** | 0.687 |
| **PC aa C32:1** | 0.458 |  | **PC aa C34:1** | 0.563 |  | **PC ae C36:2** | 0.701 |
| **PC ae C38:3** | 0.465 |  | **PC ae C34:2** | 0.565 |  | **PC ae C38:3** | 0.761 |
| **Leucine** | 0.469 |  | **Isoleucine** | 0.576 |  | **PC aa C34:1** | 0.780 |
| **PC ae C38:2** | 0.472 |  | **Valine** | 0.578 |  | **PC aa C32:1** | 0.789 |
| **PC ae C36:1** | 0.502 |  | **Leucine** | 0.580 |  | **Leucine** | 0.810 |
| **PC ae C36:2** | 0.509 |  | **PC ae C36:2** | 0.594 |  | **Isoleucine** | 0.814 |
| **PC ae C34:2** | 0.545 |  | **Ornithine** | 0.668 |  | **Valine** | 0.849 |
